# m^6^A‐dependent upregulation of DDX21 by super‐enhancer‐driven IGF2BP2 and IGF2BP3 facilitates progression of acute myeloid leukaemia

**DOI:** 10.1002/ctm2.1628

**Published:** 2024-04-04

**Authors:** Yanchun Zhao, Yutong Zhou, Yu Qian, Wenwen Wei, Xiangjie Lin, Shihui Mao, Jie Sun, Jie Jin

**Affiliations:** ^1^ Department of Hematology The First Affiliated Hospital Zhejiang University School of Medicine Hangzhou Zhejiang China; ^2^ Zhejiang Provincial Key Laboratory of Hematopoietic Malignancy Zhejiang University Hangzhou Zhejiang China; ^3^ Zhejiang Provincial Clinical Research Center for Hematological Disorders Hangzhou Zhejiang China; ^4^ Zhejiang University Cancer Center Hangzhou Zhejiang China; ^5^ Jinan Microecological Biomedicine Shandong Laboratory Jinan Shandong China

**Keywords:** acute myeloid leukaemia (AML), DDX21, N6‐methyladenosine (m^6^A), super‐enhancers (SEs)

## Abstract

**Background:**

Acute myeloid leukaemia (AML) is a haematological malignancy with unfavourable prognosis. Despite the effectiveness of chemotherapy and targeted therapy, relapse or drug resistance remains a major threat to AML patients. N6‐methyladenosine (m^6^A) RNA methylation and super‐enhancers (SEs) are extensively involved in the leukaemogenesis of AML. However, the potential relationship between m^6^A and SEs in AML has not been elaborated.

**Methods:**

Chromatin immunoprecipitation (ChIP) sequencing data from Gene Expression Omnibus (GEO) cohort were analysed to search SE‐related genes. The mechanisms of m^6^ A‐binding proteins IGF2BP2 and IGF2BP3 on DDX21 were explored via methylated RNA immunoprecipitation (MeRIP) assays, RNA immunoprecipitation (RIP) assays and luciferase reporter assays. Then we elucidated the roles of DDX21 in AML through functional assays in vitro and in vivo. Finally, co‐immunoprecipitation (Co‐IP) assays, RNA sequencing and ChIP assays were performed to investigate the downstream mechanisms of DDX21.

**Results:**

We identified two SE‐associated transcripts IGF2BP2 and IGF2BP3 in AML. High enrichment of H3K27ac, H3K4me1 and BRD4 was observed in IGF2BP2 and IGF2BP3, whose expression were driven by SE machinery. Then IGF2BP2 and IGF2BP3 enhanced the stability of DDX21 mRNA in an m^6^A‐dependent manner. DDX21 was highly expressed in AML patients, which indicated a poor survival. Functionally, knockdown of DDX21 inhibited cell proliferation, promoted cell apoptosis and led to cell cycle arrest. Mechanistically, DDX21 recruited transcription factor YBX1 to cooperatively trigger ULK1 expression. Moreover, silencing of ULK1 could reverse the promoting effects of DDX21 overexpression in AML cells.

**Conclusions:**

Dysregulation of SE‐IGF2BP2/IGF2BP3‐DDX21 axis facilitated the progression of AML. Our findings provide new insights into the link between SEs and m^6^A modification, elucidate the regulatory mechanisms of IGF2BP2 and IGF2BP3 on DDX21, and reveal the underlying roles of DDX21 in AML.

## Graphical abstract

1. In AML, two SE‐related transcripts IGF2BP2 and IGF2BP3 were identified, whose activation could be triggered by SE machinery.

2. IGF2BP2 and IGF2BP3 strengthened the stability of DDX21 mRNA in an m^6^ A‐dependent manner.

3. Knockdown of DDX21 inhibited proliferation, promoted apoptosis and led to cell cycle arrest in AML cells.

4. DDX21 recruited transcription factor YBX1 to cooperatively facilitate the transcription of ULK1.

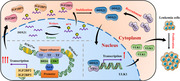

## 1 INTRODUCTION

Acute myeloid leukaemia (AML) is a malignant haematological disease characterised by impaired myeloid differentiation and abnormal proliferation of haematopoietic stem cells (HSCs).^1^ It is the most common type of adult leukaemia with a median age at diagnosis of 68 years.^2^ Intensive chemotherapy followed by consolidation chemotherapy and HSC transplantation is the major treatment. Additionally, the emerging targeted therapies show promising prospects.^3^ In AML patients, median overall survival (OS) is 8.5 months, and 5‐year OS rate is 24.0%.^4^ Due to the existence of leukaemic stem cells (LSCs) and the heterogeneity of AML, relapse or drug resistance remains a great challenge and obstacle for the survival of patients.^5^ Hence, it is urgent to investigate novel mechanisms of AML to establish more effective therapeutic strategies.

Emerging as a frontier of epigenetic modulation, RNA modifications especially N6‐methyladenosine (m^6^A) RNA methylation attract increasingly more attention. m^6^A is installed by m^6^A methyltransferases (‘writers’) such as methyltransferase‐like protein 3 (METTL3) and METTL14, and eliminated by m^6^A demethylases (‘erasers’) containing fat‐mass and obesity‐associated protein (FTO) and α‐ketoglutarate‐dependent dioxygenase alkB homologue 5 (ALKBH5).^6^ More importantly, the effects of m^6^A modification rely on different m^6^A‐binding proteins (‘readers’). As the most common readers, YT521‐B homology domain family (YTHDFs) and insulin‐like growth factor 2 mRNA‐binding proteins (IGF2BPs) are responsible for the regulation of mRNA stability and translation.^7^, ^8^ Abundant evidence demonstrates that m^6^A‐binding proteins are widely involved in various biological processes including viral replication, adipogenesis, immune response and tumorigenesis, especially AML leukaemogenesis.^8^ For example, IGF2BP2 elevated the expression of several key targets in glutamine metabolism, thereby leading to the development of AML.^9^ In addition, IGF2BP3 triggered the progression of AML via strengthening RCC2 mRNA stability.^10^ Until now, studies have predominately focused on the individual role of each m^6^A‐binding protein. Considering the analogous effects of IGF2BPs in enhancing mRNA stabilisation, there may be downstream targets synergistically regulated by them in AML.

Different from typical enhancers, super‐enhancers (SEs) are large clusters of active enhancers in proximity of 12.5 kb with one another,^11^ which cooperatively exert a strong activation on the transcription of genes determining cell identity.^12^ SEs are composed of substantial transcription factors (TFs), chromatin regulators, mediator and multiple co‐activators such as bromodomain‐containing protein 4 (BRD4), cyclin‐dependent kinase 7 (CDK7), mediator complex subunit 1 (MED1) and E1A binding protein P300 (EP300).^13^, ^14^ Several histone modifications are identified in SEs, mainly including histone H3 lysine 27 acetylation (H3K27ac) and histone H3 lysine 4 monomethylation (H3K4me1).^11^ It was noteworthy that SEs closely participated in the leukaemogenesis of AML. H3K27ac modification was increased in the SE regions of HOXA and HOXB, leading to the progression of AML.^15^ BRD4, CDK8 and other SE‐related co‐activators were also involved in AML occurrence.^13^, ^16^

Over the past few decades, the complicated crosstalk between m^6^A RNA methylation and several other epigenetic modifications has been elucidated,^17^ which plays a significant role in tumorigenesis. However, the link between m^6^A and SEs remains vague. Some possible impacts of m^6^A on SEs have been reported.^18^, ^19^ In renal cell carcinoma, FTO sustained the stability of BRD9 via mediating its demethylation, thus facilitating tumour growth.^18^ Besides, METTL14 deficiency intensified the stability of BPTF, which activated glycometabolism‐associated genes via an SE‐dependent manner, contributing to lung metastasis.^19^ Nevertheless, the specific roles of SEs on m^6^A modification have not been elaborated, especially in AML.

DEAD box proteins characterised by the conserved motif Asp‐Glu‐Ala‐Asp are identified as RNA helicases. DEAD box protein 21 (DDX21) is located in nucleus, regulating ribosomal RNA (rRNA) biogenesis.^20^ Moreover, DDX21 promotes gene transcription via reducing the formation of R‐loop,^21^ extensively participating in tumorigenesis and antiviral immunity.^22^, ^23^ In AML1‐ETO‐positive AML, amino‐terminal enhancer of split (AES) induced snoRNA/RNP formation with the assistance of DDX21, leading to leukaemogenesis.^24^ However, definite functions and mechanisms of DDX21 in the pathogenesis of AML remain unclear.

In the current study, IGF2BP2 and IGF2BP3 were identified as two SE‐related transcripts in AML. The expression of IGF2BP2/IGF2BP3 was driven by SEs. As m^6^A‐binding proteins, IGF2BP2 and IGF2BP3 strengthened the stability of DDX21 in an m^6^A‐dependent manner. DDX21 was highly expressed in AML, indicating an unfavourable prognosis. Functionally, knockdown of DDX21 suppressed cell proliferation, facilitated cell apoptosis and led to cell cycle arrest in vitro, and inhibited AML progression in vivo. Mechanistically, DDX21 triggered the transcription of ULK1 via recruiting transcription factor YBX1. These findings enrich the understanding of the relationship between SEs and m^6^A modification, shed light on the common mechanisms of IGF2BP2/IGF2BP3 on DDX21, and emphasise the meaningful values of DDX21 in AML progression.

## 2 METHODS

### 2.1 Cell culture

Human leukaemia cell lines MOLM13 and THP‐1 were cultured in RPMI‐1640 medium (Gibco) supplemented with 10% fetal bovine serum (FBS, Gibco) and incubated at 37°C in a humidified atmosphere containing 5% CO_2_. All cell lines were validated with STR profiling and free of mycoplasma contamination.

### 2.2 Primary samples

All samples from newly diagnosed AML or non‐AML patients were collected from bone marrow aspirations after obtaining informed consent. Then mononuclear cells (MNCs) were isolated for subsequent experiments. Peripheral blood mononuclear cells (PBMCs) from healthy donors were purified using Ficoll. All experiments using human specimens were approved by the Institutional Review Board (IRB) of the First Affiliated Hospital, School of Medicine, Zhejiang University. And the IRB number was 2023‐0625.

### 2.3 Cell transfection

Knockdown and overexpression plasmids were constructed by RiboBio Co., Ltd. Lentiviruses were generated with the help of HEK‐293T cells, and then transfected into MOLM13 and THP‐1 cells, respectively. Cells were selected using 1 µg/mL puromycin (MCE), and the efficiency was determined by RT‐qPCR and Western blotting assays for subsequent experiments. All targeted sequences of shRNA are showed in Table S1.

### 2.4 Quantitative reverse transcription PCR (RT‐qPCR)

Total RNAs were extracted from cells via RNA‐Quick Purification Kit (YiShan Biotechnology), and then reversely transcribed into cDNA using HiScript II Q RT SuperMix for qPCR (Vazyme). qPCR with ChamQ SYBR qPCR Master Mix (Vazyme) was performed to measure the expression of RNAs with the assistance of QuantStudio 5 Real‐Time PCR System (Thermo Fisher Scientific). 2^ΔΔCt^ method was applied to calculate the relative expression of RNAs. The primers were constructed by Tsingke Biotechnology, and are listed in Table S2.

### 2.5 Western blotting

Total proteins were isolated from cells using RIPA buffer (Beyotime). The collected supernatant was subjected to quantification with the aid of Bicinchoninic Acid Protein Assay Kit (Thermo Fisher Scientific). Proteins were segregated with 4%–20% SDS‐PAGE (GenScript) and transferred into.22 µm PVDF membranes (Millipore), followed by the incubation in 5% non‐fat milk for 1 h. The membranes were then incubated in primary antibodies at 4°C overnight. The next day, after being washed at least three times, the membranes were incubated in secondary antibodies at room temperature for 1 h. The imaging system (Clinx) was applied to detect the immunoblots using enhanced chemiluminescence kit (FDbio science). The antibodies are shown in Table S3.

### 2.6 Chromatin immunoprecipitation (ChIP) assay

ChIP assays were carried out with SimpleChIP Enzymatic Chromatin IP Kit (Magnetic Beads) (Cell Signaling Technology) according to the protocols of manufacturer. In brief, approximately 1 × 10^7^ cells were prepared and fixed with 37% formaldehyde at a final concentration of 1%. The collected cell precipitations were treated with micrococcal nuclease and incubated at 37°C for 20 min to digest DNA, followed by the sonication. Then the immunoprecipitating antibodies (Table S3) were added into the supernatant with the rotation at 4°C overnight. The next day, Protein G Magnetic Beads were subjected to the above mixture and rotated at 4°C for 2 h. After being washed, 5 M NaCl and proteinase K were added with the rotation at 65°C for another 2 h. Finally, DNAs in the immunoprecipitation were purified using elution buffers for subsequent qPCR assays. Primers are included in Table S2.

### 2.7 ChIP‐seq data analysis

ChIP‐seq data in MOLM14 cells, including H3K27ac, H3K4me1 and BRD4, were obtained from Gene Expression Omnibus (GEO) cohort GSE65161 (including GSM1587891, GSM1587892, GSM1587893, GSM1587908, GSM1587909, GSM1587910, GSM1587911, GSM1587904, GSM1587905, GSM1893934, GSM1893935, GSM1893936 and GSM1893937). We performed the analysis of identifying SE and its associated genes with the assistance of RiboBio Co., Ltd. Constituent enhancers that occurred within 12.5 kb were further stitched together for SE identification by Rank Ordering of Super Enhancers (ROSE) algorithm.^11^, ^12^, ^25^ According to the H3K27ac, H3K4me1 and BRD4 ChIP‐Seq signals, enhancer regions were plotted in an increasing order to form the hockey stick plots. Enhancers that were located above the inflection point of the curve were defined as SEs.^11^ SEs were assigned to genes with TSS flanking a 50‐kb window of the SEs.

### 2.8 DNA gel electrophoresis

1.5% Agarose (Solarbio) was dissolved in .5×TBE (Solarbio) under a heating condition, and then cooled to form a gel. The products of RT‐qPCR assays and 6× loading buffer were mixed and added into the pores of gel, which was placed in the electrophoresis tank (Bio‐RAD) filled with .5×TBE. Then the electrophoresis tank worked at 130 V for 40 min. The results were obtained via an imaging system (Proteinsimple).

### 2.9 Methylated RNA immunoprecipitation (MeRIP) assay

MeRIP assays were conducted with Magna MeRIP m^6^A Kit (Millipore). At least 200 µg total RNA was prepared and fragmented into less than 100 nucleotides. 3 M sodium acetate, glycogen and absolute ethanol were added and subsequently stored at −80°C overnight. Afterwards, Magna ChIP Protein A/G Magnetic Beads with 10 µg IgG or m^6^A antibody were subjected to fragmented RNAs with the rotation at 4°C for 2 h. After the beads were washed, the precipitated RNAs were eluted for RT‐qPCR assays. The primers were designed based on the predicted m^6^A sites on DDX21 from SRAMP software (http://www.cuilab.cn/sramp) and RMBase v2.0 (http://rna.sysu.edu.cn/rmbase/), and are given in Table S2.

### 2.10 RNA immunoprecipitation (RIP) assay

According to the manufacturer's guidance, Magna RIP RNA‐Binding Protein Immunoprecipitation Kit (Millipore) was applied to carry out RIP assays. RIP lysis buffers with protease inhibitor and RNase inhibitor was added into cells and then stored at −80°C overnight. Cell lysates were subjected to the beads with corresponding antibodies (Table S3) or IgG, and incubated at 4°C overnight. The next day, proteinase K buffers were added and the mixture was shaken at 55°C for 30 min to digest the proteins. The RNAs of precipitated complex were eluted for subsequent RT‐qPCR assays. Primers are listed in Table S2.

### 2.11 Co‐immunoprecipitation (Co‐IP) assay

Dynabeads Co‐Immunoprecipitation Kit (ThermoFisher Scientific) was applied to conduct Co‐IP assays. The mixture of beads and antibodies (Table S3) was rolled at 37°C overnight. Afterwards, the collected cell lysates were added into the beads and rotated at 4°C for 30 min to form an immunoprecipitated protein complex. After the beads were washed, the binding proteins were eluted for subsequent Western blotting assays.

### 2.12 Construction of truncation plasmids for IGF2BP2 and IGF2BP3

IGF2BP2 and IGF2BP3 consisted of four KH domains and two RRM domains. To investigate the binding sites of IGF2BP2 and IGF2BP3 to DDX21, wild‐type (WT) and truncation mutant plasmids were constructed by RiboBio Co., Ltd. All plasmids were equipped with flag‐tag. The corresponding sequences are exhibited inSupporting Information.

### 2.13 Luciferase reporter assay

The DNA fragments of mutant reporter plasmids were constructed through replacing the adenine (A) at the predicted m^6^A sites of DDX21 to cytosine (C). WT and mutant were inserted into downstream of firefly luciferase of pMIR‐REPORT vector (test plasmids). For dual‐luciferase reporter assay, 500 ng test plasmids and 50 ng pRL‐TK (renilla luciferase control reporter vector) were co‐transfected into HEK‐293T cells with IGF2BP2 or IGF2BP3 silencing in 24‐well plates. After 48 h, luciferase activities of cells were detected with Dual Luciferase Reporter Assay Kit (Vazyme). The results were calculated by the ratio of luciferase activities between firefly and renilla. The corresponding sequences of reporter plasmids are shown inSupporting Information.

### 2.14 RNA decay assay

The cells with IGF2BP2 or IGF2BP3 knockdown or not were seeded into six‐well plates. Then actinomycin D (Selleck) was added into cells with a final concentration of 5 µg/mL. After incubation for 0, 2 and 4 h, cells were successively harvested to extract the total RNAs for subsequent RT‐qPCR assays.

### 2.15 CCK‐8 assay

A total of 1 × 10^4^ cells were seeded into 96‐well plates. After incubation for 0, 24, 48, 72 and 96 h, cell counting kit‐8 (CCK‐8, MCE, HY‐K0301) was added, respectively. Absorbance at 450 nm was measured via spectrophotometer (BioTek) after cells were incubated with CCK‐8 for 1−2 h at 37°C.

### 2.16 Apoptosis assay

Annexin V‐APC/PI apoptosis kit (MULTI sciences) was applied to detect the apoptosis of cells. The collected cells were washed by pre‐cooled PBS, followed by the resuspension with 1× binding buffer. Then the cells were stained with Annexin V and PI, and incubated at room temperature for 5 min before being measured via flow cytometer (ACEA Biosciences, Inc.).

### 2.17 5‐Ethynyl‐2′‐deoxyuridine (EdU) assay

EdU assays were carried out with BeyoClick EdU Cell Proliferation Kit with Alexa Fluor 555 (Beyotime) according to the manufacturer's instructions. Cells were incubated with EdU at 37°C for 2 h, followed by the successive treatments with 4% paraformaldehyde and permeable fluid. Then cells were incubated away from light for 30 min at room temperature with Click reaction solution. The DNA replication abilities of cells were detected by flow cytometer (ACEA Biosciences, Inc.).

### 2.18 Cell cycle assay

The collected cells were fixed with pre‐cooled 75% ethanol and stored at −20°C overnight. After ethanol was discarded, cells were hydrated with PBS for 15 min, and then incubated with 300 µL DNA staining solution (MULTI sciences) at room temperature for 30 min. Cell cycle analysis was performed by flow cytometer (ACEA Biosciences, Inc.).

### 2.19 Silver staining assay and mass spectrometry (MS) analysis

After immunoblotting of proteins obtained from Co‐IP assays with DDX21 and IgG antibodies, silver staining assays were performed using Fast Silver Stain Kit (Beyotime) according to the protocols. The differential proteins between IgG and DDX21‐IP groups were observed according to the intensity of silver staining. Then the corresponding gel was further subjected to MS analysis (OE Biotech Co., Ltd).

### 2.20 RNA sequencing

MOLM13 cells with DDX21 knockdown (sh1 and sh3) and negative control were prepared to extract the total RNAs. RNA sequencing (RNA‐seq) was accomplished by OE Biotech Co., Ltd with Illumina NovaSeq 6000 (Illumina). Differential expression genes were analysed with the criteria of *q* <05 and fold change (FC) >2 or <.5.

### 2.21 Xenograft models

Five‐week‐old female NCG mice were purchased from GemPharmatech Co., Ltd and randomly divided into three groups. Then 1 × 10^6^ luciferase‐labelled THP‐1 cells with DDX21‐knockdown (sh1 and sh3) or negative control were injected into three groups of mice via tail vein, respectively. The tumour load of mice was monitored via IVIS Imaging System after luciferin (Promega) intraperitoneal injection every week. All animal experiments were approved by the Ethics Committee on Animal Experiments of the First Affiliated Hospital of Zhejiang University and performed in accordance with ethical guidelines.

### 2.22 Statistical analysis

All experiments were independently repeated at least three times. Measured data were analysed using GraphPad Prism 8.0 software and shown as mean ± standard deviation (SD). The comparison of quantitative data was conducted via two‐tailed Student's *t*‐test. Kaplan–Meier analysis with log‐rank test was performed to evaluate the survival. The linear regression was applied to analyse the correlation between two genes. The *p*‐value considered statistically significant was less than.05 (*p < .05, **p < .01, ***p < .001, ****p < .0001).

## 3 RESULTS

### 3.1 IGF2BP2 and IGF2BP3 were identified as SE‐driven transcripts

**FIGURE 1:**
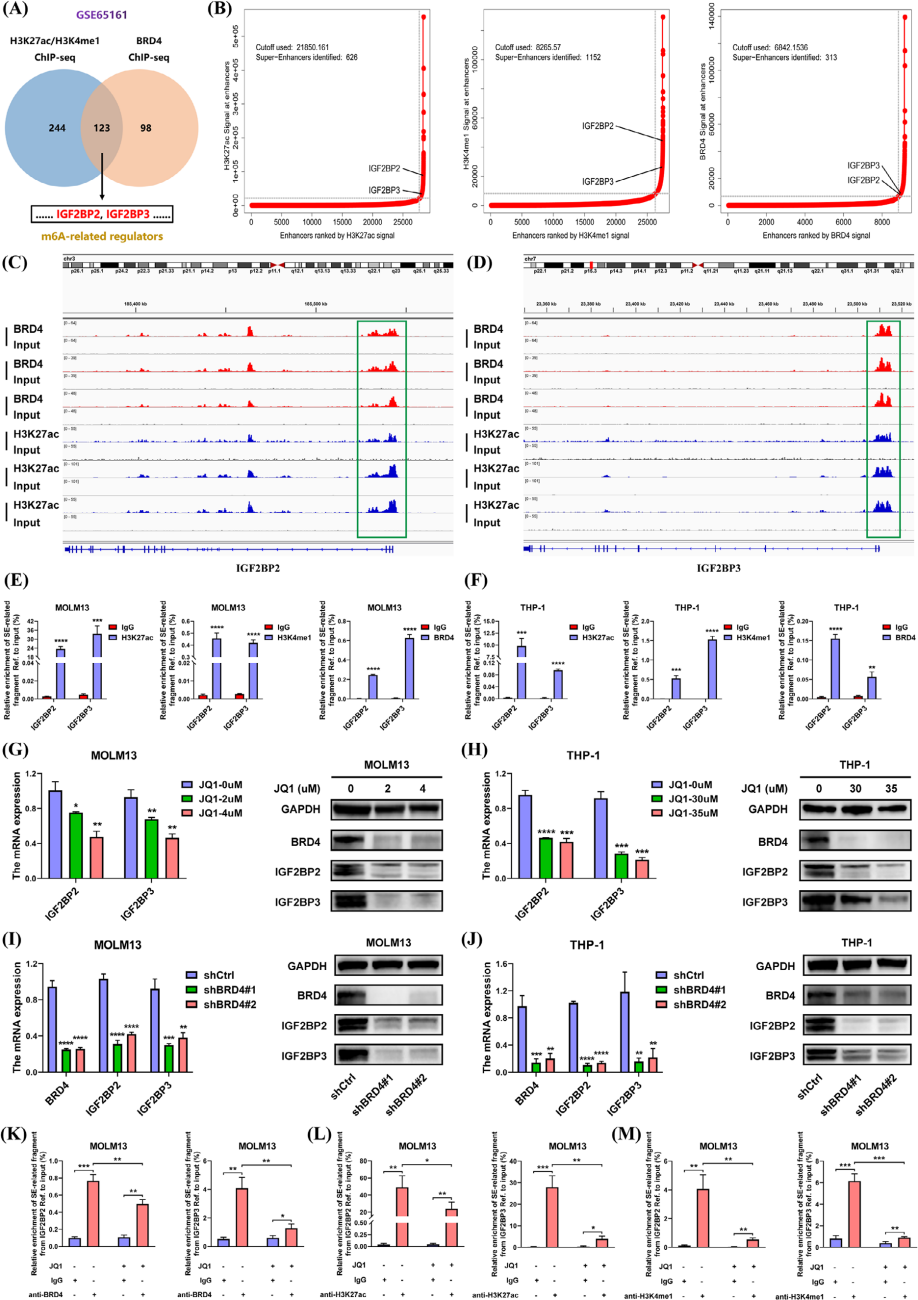
SEs participated in the regulation of IGF2BP2 and IGF2BP3. (A) A Venn diagram was generated to present the SE‐associated genes based on the ChIP‐seq data of H3K27ac, H3K4me1 and BRD4 in MOLM14 cells (GSE65161). Two m^6^A readers, IGF2BP2 and IGF2BP3, were found at the intersection. (B) Hockey stick plots depicted the rank order of enhancers based on H3K27ac (left), H3K4me1 (middle) and BRD4 (right) signals (GSE65161). SEs were defined as the enhancers that were located above the inflection point of curve, thus IGF2BP2 and IGF2BP3 were considered as two SE‐related genes. (C and D) Integrative Genomics Viewer (IGV) tracks of BRD4 and H3K27ac signals displayed the SE regions on IGF2BP2 (C) and IGF2BP3 (D) where peaks were notably enriched compared with the input. (E and F) ChIP‐qPCR assays were performed in MOLM13 (E) and THP‐1 (F) cells to confirm the enrichment of H3K27ac, H3K4me1 and BRD4 on IGF2BP2 and IGF2BP3. (G and H) RNA (left) and protein (right) levels of IGF2BP2 and IGF2BP3 were detected after the treatment of JQ1. (I and J) The inhibitory effects of BRD4 knockdown on the expression of IGF2BP2 and IGF2BP3 were assessed by RT‐qPCR (left) and Western blotting (right) assays. (K–M). MOLM13 cells were treated with or without JQ1 for 24 h. ChIP assays with antibodies against BRD4 (K), H3K27ac (L) or H3K4me1 (M) were performed, respectively. The alterations of BRD4, H3K27ac and H3K4me1 enrichment on IGF2BP2 and IGF2BP3 after JQ1 treatment were assessed by subsequent qPCR assays (*p < .05, **p < .01, ***p < .001, ****p < .0001).

Increasing evidence has demonstrated that m^6^A RNA methylation is closely relevant to the pathogenesis of AML. However, more attention has been attracted on the downstream mechanisms rather than upstream machinery of m^6^A modification in AML. SEs predominantly function to strengthen transcription processes, which play a crucial role in the gene expression. To investigate the link between SEs and m^6^A, we analysed the publicly available ChIP sequencing (ChIP‐seq) data of three major active enhancer markers H3K27ac, H3K4me1 and BRD4 in MOLM14 cells from GEO cohort (GSE65161), and found 123 SE‐associated genes (Table S4). Intriguingly, two m^6^A‐binding proteins IGF2BP2 and IGF2BP3 were observed in the list (Figure 1A). Whether IGF2BP2 and IGF2BP3 are modulated by SE machinery requires further verification.

We systematically profiled the landscape of these three SE‐associated markers and characterised IGF2BP2 and IGF2BP3 as SE‐related transcripts based on H3K27ac, H3K4me1 and BRD4 signals (Figure 1B). Moreover, the visible peaks of BRD4 and H3K27ac were observed in the SE regions of IGF2BP2 and IGF2BP3 (Figure 1C,D). Our ChIP‐qPCR assays also verified the increased enrichment of H3K27ac, H3K4me1 and BRD4 on IGF2BP2 and IGF2BP3 in AML cells (Figure 1E,F and Figure S1A–D). Subsequently, we divided the SE regions of IGF2BP2 and IGF2BP3 into two constituents E1 and E2, respectively. Three luciferase reporters including Promoter, Enhancer#1 and Promoter and Enhancer#2 and Promoter were constructed for luciferase reporter assays (Figure S1E, and the corresponding sequences shown in Supporting Information). As a result, strong transcription‐enhancing activity was observed in cells transfected with Enhancer and Promoter plasmids (Figure S1F,G).

It is reported that bromodomain and extra‐terminal (BET) inhibitor JQ1 can lead to the loss of BRD4 at SEs and reduce the transcription elongation of SE‐driven genes.^26^ After AML cells were treated with JQ1, the expressions of IGF2BP2 and IGF2BP3 were both decreased (Figure 1G,H), while the expressions of METTL3, METTL14, FTO and ALKBH5 were not altered (Figure S1H,I). Moreover, knockdown of BRD4 also triggered the suppression of IGF2BP2 and IGF2BP3 (Figure 1I,J). More importantly, we observed that the occupancy of BRD4, H3K27ac and H3K4me1 at the SE regions of IGF2BP2 and IGF2BP3 could be blocked by JQ1 treatment (Figure 1K–M). Additionally, CDK7 inhibitor THZ1 was also able to diminish the levels of IGF2BP2 and IGF2BP3 (Figure S1J,K). To sum up, IGF2BP2 and IGF2BP3 are two SE‐driven transcripts.

### 3.2 SE‐driven IGF2BP2 and IGF2BP3 were involved in the regulation of DDX21 via an m^6^A‐dependent manner

**FIGURE 2:**
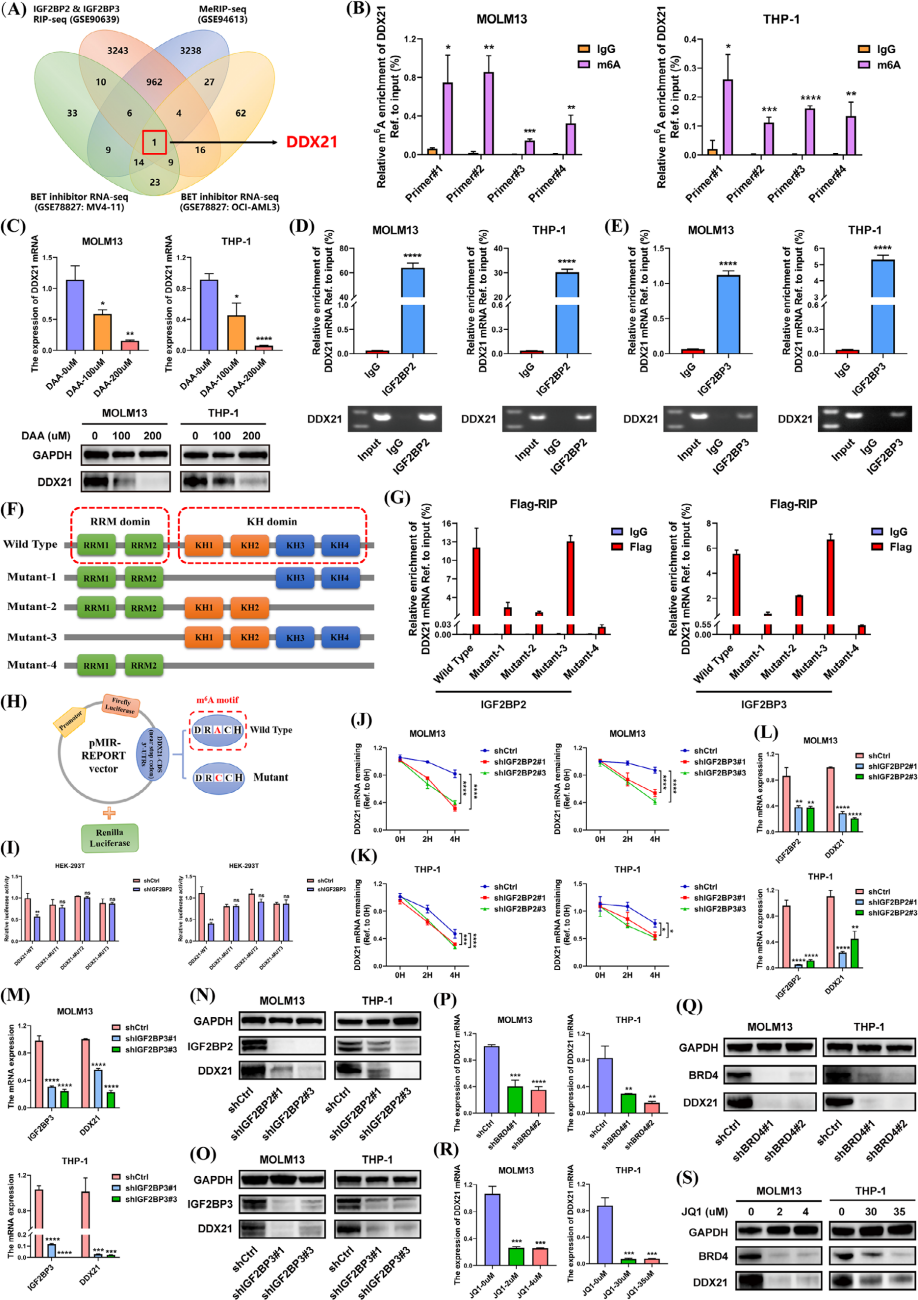
m^6^A‐modified DDX21 was modulated by SE‐driven IGF2BP2 and IGF2BP3. (A) A Venn diagram based on three GEO datasets was employed to explore the possible downstream transcripts of IGF2BP2 and IGF2BP3. The data were obtained from IGF2BP2/IGF2BP3 RIP‐seq in HEK‐293T cells (GSE90639), MeRIP‐seq in MOLM13 cells (GSE94613) and BET inhibitor RNA‐seq in MV4‐11 and OCI‐AML3 cells (GSE78827). In GSE78827, RNA‐seq data of AML cells treated with DMSO or JQ1 were selected to identify the transcripts suppressed by JQ1 (*p* < .05 and FC < .5). (B) MeRIP assays with m^6^A‐specific antibodies were performed to determine the m^6^A abundance on DDX21. Relative m^6^A enrichment was obtained by comparing m^6^A‐IP/input with IgG‐IP/input. (C) MOLM13 and THP‐1 cells were treated with DAA in different concentrations (0, 100 and 200 µM, respectively). The alterations of DDX21 expression were detected. (D and E) The binding of IGF2BP2 (D) or IGF2BP3 (E) to DDX21 was identified by RIP assays. Results of RT‐qPCR and DNA gel electrophoresis assays are exhibited. (F) As the schematic illustration shows, four truncation mutants (M1–4) and one wild‐type (WT) plasmid with flag‐tag were constructed for IGF2BP2 and IGF2BP3, respectively. (G) Above plasmids were separately transfected into HEK‐293T cells. RIP assays with IgG or flag antibodies were performed to determine the domains binding to DDX21. (H) The m^6^A sites of DDX21 were predicted to be mainly located at the CDS near stop codon and 3′UTRs according to the m^6^A motif DRACH. In mutant plasmid, adenosine (A) bases in possible m^6^A sites were replaced by cytosine (C) bases. (I) These plasmids mentioned above were transfected into HEK‐293T cells whose IGF2BP2 or IGF2BP3 was stably knocked down, respectively. Following luciferase reporter assays were carried out. (J and K) The impacts of IGF2BP2 or IGF2BP3 on DDX21 stability were investigated via RNA decay assays with actinomycin D. (L–O) The expression of DDX21 was measured when IGF2BP2 or IGF2BP3 was suppressed. (P–S) After the loss of BRD4 (P and Q) or the treatment of JQ1 (R and S), DDX21 expression was examined (*p < .05, **p < .01, ***p < .001, ****p < .0001).

Considering that IGF2BP2 and IGF2BP3 were not only essential m^6^A readers, but also activated by SEs, we integrated three GEO datasets to shed light on the downstream targets of SE‐IGF2BP2/IGF2BP3 axis in AML. RIP‐seq of IGF2BP2 and IGF2BP3 (GSE90639) showed the common binding targets of them. MeRIP‐seq (GSE94613) exhibited m^6^A‐modifed genes regulated by METTL3. RNA‐seq of BET inhibitors (GSE78827) presented the transcripts suppressed by JQ1 (Figure 2A). Surprisingly, DDX21 was the sole gene in the overlap. Hence, DDX21 may be a vital target of IGF2BP2/IGF2BP3 under the regulation of SEs and m^6^A machinery.

To validate the hypothesis, we first performed MeRIP‐qPCR assays to determine whether DDX21 was modified by m^6^A (Figure S2A). Compared with IgG control, m^6^A‐specific antibody had a remarkable enrichment at the potential m^6^A sites on DDX21 (Figure 2B and Figure S2B,C). When METTL3 was silenced, the m^6^A level on DDX21 was diminished (Figure S2D) and the expression of DDX21 was reduced (Figure S2E,F), which verified the results of MeRIP‐seq from GSE94613. In addition, after AML cells were treated with the methylation inhibitor 3‐deazaadenosine (DAA), the expression of DDX21 was decreased (Figure 2C). These findings suggested that DDX21 was an m^6^A‐modifed gene.

Subsequently, RIP assays confirmed that both IGF2BP2 and IGF2BP3 could bind to DDX21 (Figure 2D,E). The structures of IGF2BPs family consisted of two RNA recognition motif (RRM) domains and four K homology (KH) domains, which serve as RNA‐binding domains recognising m^6^A sites on mRNAs. In order to map the specific domains of IGF2BP2 and IGF2BP3 responsible for binding to DDX21, four flag‐tagged truncation mutant plasmids were constructed (mutants 1–4 labeled as M1–4, respectively, and the corresponding sequences shown in Supporting Information; Figures 2F and S2G,H). The enrichment of DDX21 in M1, M2 and M4 groups were significantly decreased compared with WT, among which M4 group showed the lowest DDX21 affinity (Figure 2G). Nevertheless, enrichment in M3 group was analogous to WT group (Figure 2G), suggesting that M3 mutant could efficiently capture DDX21 mRNA. These results indicated that KH1–4 domains of IGF2BP2 and IGF2BP3 were indispensable for the interaction with DDX21.

To further illustrate the significance of m^6^A for DDX21, WT and mutant plasmids were designed for luciferase reporter assays. Different from WT group, the predicted m^6^A sites of DDX21 were mutated to impede m^6^A methylation in mutant reporters (Figure 2H and Figure S2I; the corresponding sequences exhibited in Supporting Information). The luciferase activities of cells in WT group attenuated when IGF2BP2 or IGF2BP3 was knocked down, while it remained unvaried in mutant groups (Figure 2I). Functionally, IGF2BP2 and IGF2BP3 strengthened the mRNA stability of DDX21 (Figure 2J,K and Figure S2J). Additionally, IGF2BP2 or IGF2BP3 deficiency resulted in the reduction of DDX21 expression, while their overexpression increased the expression of DDX21 (Figure 2L–O and Figure S3A–D). Notably, silencing IGF2BP2 did not influence the IGF2BP3 expression, and vice versa (Figure S3E–H). It indicated that IGF2BP2 and IGF2BP3 played a cooperative role in the regulation of DDX21.

As IGF2BP2 and IGF2BP3 were dominated by SEs, we also investigated the impacts of SEs on DDX21. Either BRD4 knockdown or SEs inhibition by JQ1 and THZ1 was able to negatively regulate DDX21 (Figure 2P–S and Figure S3I,J). Besides, rescue assays affirmed that overexpression of IGF2BP2 or IGF2BP3 was sufficient to abrogate the suppression of DDX21 owing to BRD4 silencing (Figure S4A–D) or JQ1 treatment (Figure S5A–D). These findings implied that DDX21 is regulated by SE‐driven IGF2BP2/IGF2BP3 in an m^6^A‐based manner.

### 3.3 DDX21 promoted proliferation of AML cells

**FIGURE 3:**
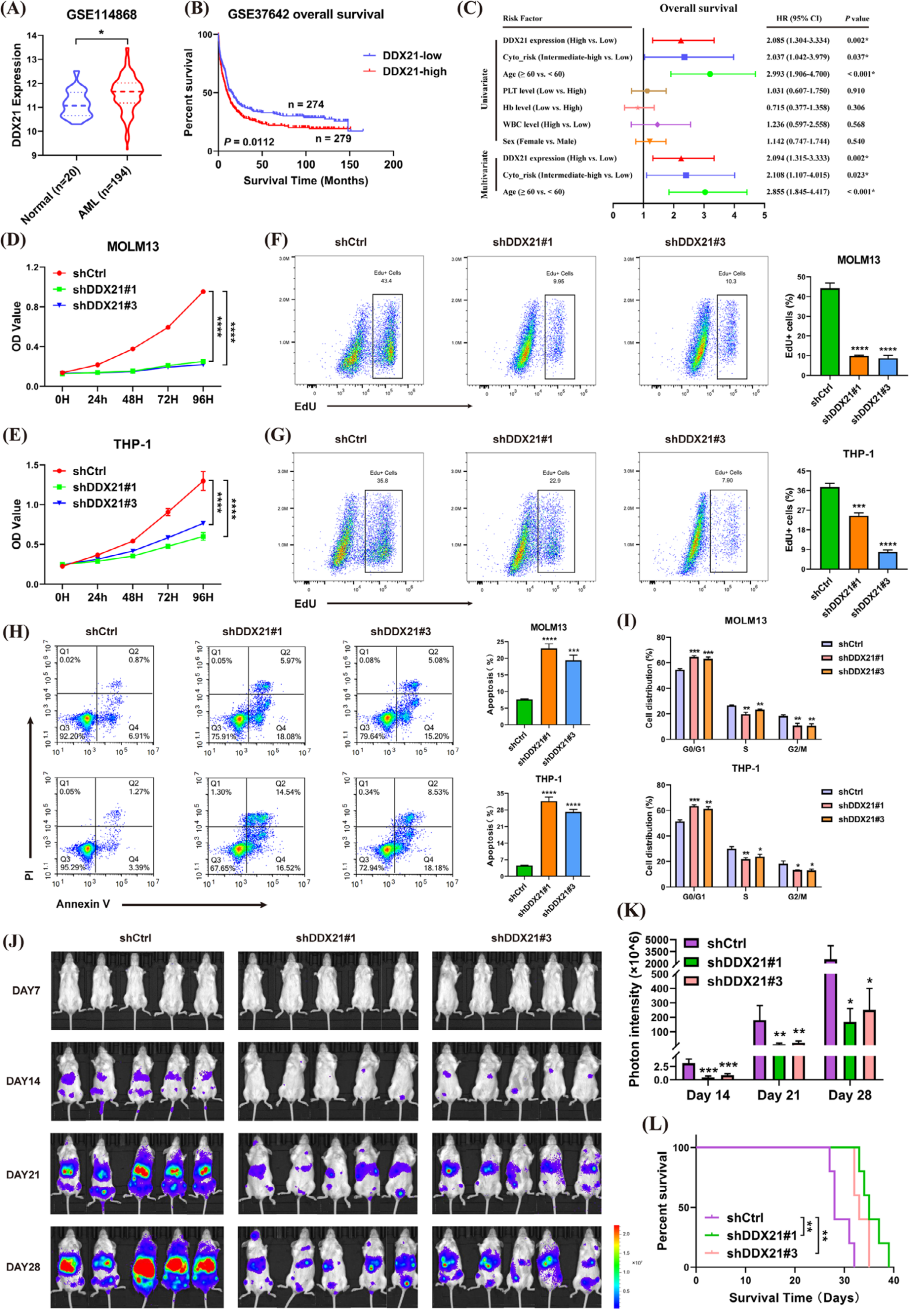
Inhibition of DDX21 suppressed the proliferation and promoted the apoptosis of AML cells. (A) The expression of DDX21 was compared between normal (*n* = 20) and AML (*n* = 194) patients from the GEO cohort (GSE114868). (B) The Kaplan–Meier survival analysis was performed in AML patients based on the expression of DDX21 from the GEO cohort (GSE37642). (C) Forest plots based upon the outcomes of COX univariate and multivariate analysis of several factors associated with OS in AML patients from TCGA database. (D and E) The proliferation abilities of MOLM13 (D) and THP‐1 (E) cells were assessed by CCK‐8 assays when DDX21 was silenced. (F and G) EdU assays were performed followed by flow cytometric analysis to assess the changes in DNA replication capabilities of cells after DDX21 was repressed (left). The bar charts show the percentages of EdU‐positive cells (right). (H) AML cells with DDX21 knockdown or not were stained with Annexin V and propidium iodide (PI), and subsequently subjected to flow cytometric analysis for apoptosis measurement (left). The percentages of apoptotic cells (Annexin V^+^) were calculated and exhibited in the bar charts (right). (I) The results of changes in cell cycle after DDX21 knockdown are shown. (J–L) Luciferase‐labelled THP‐1 cells were transfected with DDX21 deficiency (sh1 and sh3) or negative control lentivirus, and then implanted into immunodeficient mice via tail vein injection (*n* = 5 for each group). The tumour load in each group of mice was monitored by IVIS at Days 7, 14, 21 and 28 (J). The photon intensities were calculated and shown in the bar chart (K). The survival of mice was evaluated by Kaplan–Meier analysis (L) (*p < .05, **p < .01, ***p < .001, ****p < .0001).

To unveil the biological roles of DDX21 in AML, we first analysed its expression utilising publicly available datasets. Compared with healthy control, DDX21 expression was increased in AML patients (Figure 3A and Figure S6A,B). In primary cells, the expression of DDX21 was also higher in AML patients than that in normal or non‐AML patients (Figure S6C). Moreover, elevated DDX21 expression denoted poor prognosis in AML (Figure 3B and Figure S6D), which was an independent prognostic factor for OS (*p* = .002) (Figure 3C and Table S5). Hence, we speculate that DDX21 may serve as a pro‐tumorigenic gene in AML.

The efficiency of DDX21 knockdown was validated before functional experiments (Figure S6E). Cell viability assays indicated that DDX21 deficiency inhibited the growth of AML cells (Figure 3D,E). As evidenced by restrained DNA replication capabilities of cells, loss of DDX21 led to the suppression of cell proliferation (Figure 3F,G). Moreover, cell apoptosis was activated when DDX21 was silenced (Figure 3H). Then the expression of apoptosis‐related proteins was examined. DDX21 knockdown facilitated the expression of cleaved‐caspase‐3 (cle‐caspase‐3) and cleaved‐PARP (cle‐PARP), which meant increased apoptosis (Figure S6F). Additionally, DDX21 deficiency resulted in a significant G0/G1 arrest (Figure 3I and Figure S6G). To evaluate the functions of DDX21 in vivo, immunodeficient mice were injected with luciferase‐labelled THP‐1 cells to establish xenograft models. The tumour load of mice was substantially reduced when DDX21 was knocked down (Figure 3J,K and Figure S6H). Meanwhile, inhibition of DDX21 remarkably prolonged survival of recipient mice (Figure 3L).

In addition, we found that the expression of IGF2BP2 or IGF2BP3 was positively correlated with DDX21 in AML patients based on TCGA and TARGET databases (Figure S7A,B). The prognostic values of DDX21 combined with IGF2BP2 or IGF2BP3 levels were also analysed. AML patients with IGF2BP2^high^DDX21^high^ or IGF2BP3^high^DDX21^high^ had a worse prognosis than any other groups, especially comparing with those belonging to IGF2BP2^low^DDX21^low^ or IGF2BP3^low^DDX21^low^ group (Figure S7C–F). In summary, DDX21 exerts an oncogenic role in AML.

### 3.4 DDX21 triggered ULK1 expression via recruiting transcription factor YBX1

**FIGURE 4:**
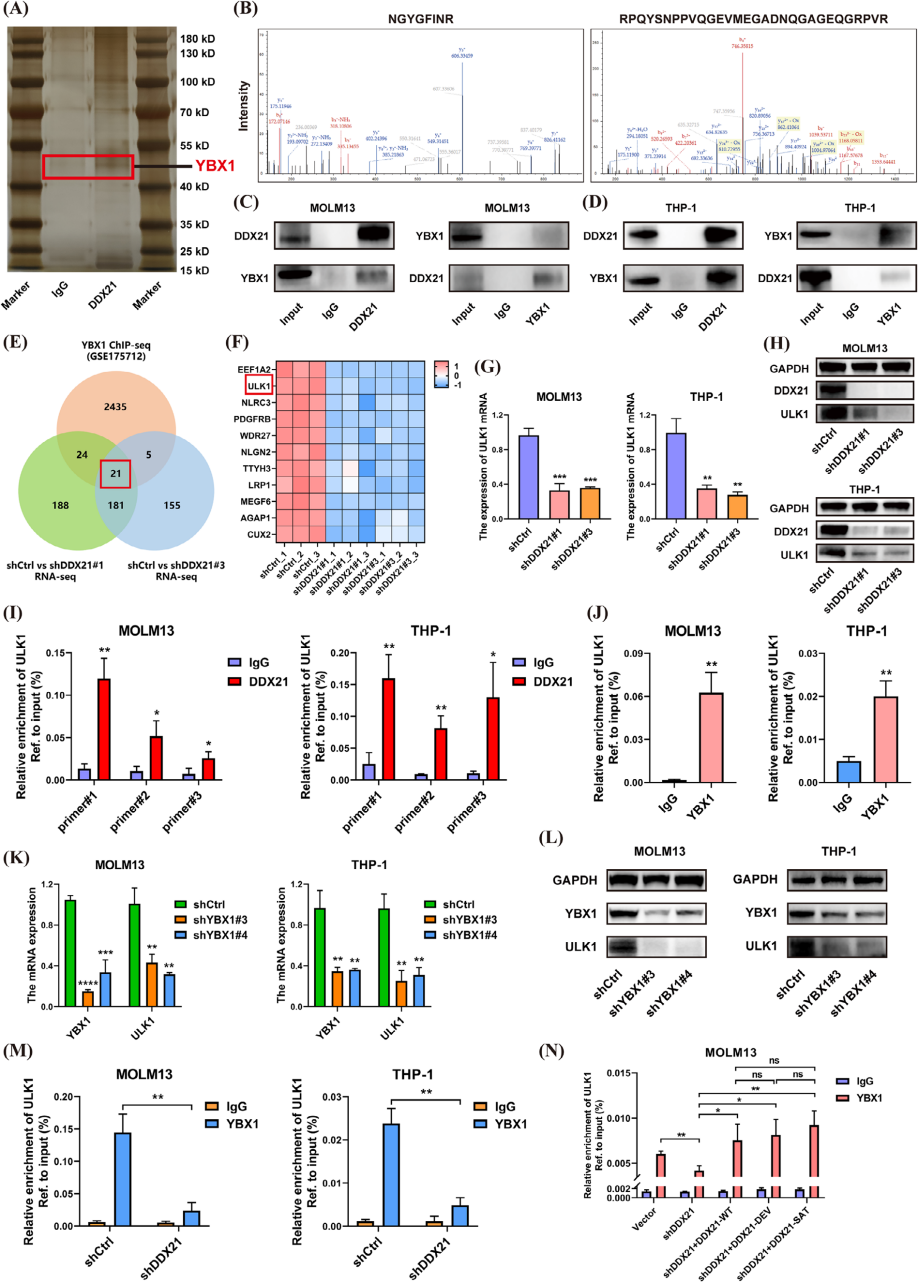
YBX1 was recruited by DDX21 to impel the transcription of ULK1. (A and B) Co‐IP assays were conducted with DDX21 and IgG antibodies, followed by the silver staining assays (A). The gels with differential staining between these two groups were applied to MS analysis, which identified two unique peptides of YBX1 (B). (C and D) Reciprocal Co‐IP assays with either DDX21 or YBX1 antibody were employed to determine whether DDX21 could interact with YBX1. (E) RNA‐seq was carried out using MOLM13 cells with DDX21 knockdown or negative control. The genes that were downregulated when DDX21 was silenced were selected for subsequent investigation (average FPKM in negative control group > 1, FC < .5, *q* < .05). Results of ChIP‐seq in GSE175713 identified the transcripts bound by YBX1. Then a Venn diagram was generated based on these two datasets. There were 21 genes in the overlap. (F) This heatmap depicted the relative expression of 11 candidate transcripts between negative control and DDX21‐knockdown groups according to our RNA‐seq data. ULK1 was one of the differential genes. (G and H) The effects of DDX21 on ULK1 expression were verified via RT‐qPCR and Western blotting assays (I and J) ChIP‐qPCR assays were applied to substantiate the occupancy of DDX21 (I) or YBX1 (J) on ULK1. Relative enrichment was normalised to the input. (K and L) The expression of ULK1 was examined when YBX1 was deficient. (M) To investigate the effects of DDX21 on YBX1‐mediated transcriptional activation, ChIP‐qPCR assays were utilised to measure the enrichment of YBX1 on the promoter of ULK1 when DDX21 was silenced or not. (N) Enrichment of YBX1 on ULK1 promoter was determined when mutated DDX21 plasmids were transfected into DDX21‐silenced cells (*p < .05, **p < .01, ***p < .001, ****p < .0001).

It is well characterised that DDX21 functions as an RNA helicase to regulate the gene transcription. In this process, there may be several transcription factors interacting with DDX21. Therefore, we performed Co‐IP assays using DDX21 antibody in AML cells. The results of silver staining showed differential imprints between DDX21‐IP and IgG groups (Figure 4A). A variety of potential binding proteins of DDX21 were obtained via MS analysis, among which RPL3, YBX1 and RPS3A were the most differential proteins. RPL3 and RPS3A were genes encoding ribosomal proteins, while YBX1 was recognised as a transcription factor whose increased expression was also an adverse prognostic factor in AML patients (Figure S8A–C). Besides, the unique peptides of YBX1 were identified in MS results (Figure 4B). Thus, YBX1 may be the most possible binding protein of DDX21. Subsequent mutual Co‐IP assays validated an interaction between DDX21 and YBX1 (Figure 4C,D).

To further determine the specific targets of DDX21 and YBX1, we analysed the ChIP‐seq of YBX1 (GSE175713) and our own RNA‐seq using DDX21‐knockdown AML cells. There were 21 genes in the overlap (Figure 4E). The survival analysis of these candidate transcripts was preformed based on TCGA datasets. A total of 11 genes were prioritised owing that their elevated expression suggested unfavourable survival of AML patients (Figure S8D–N). As the heatmap exhibited, these 11 transcripts were downregulated when DDX21 was silenced (Figure 4F). Then AML cells with DDX21 deficiency were constructed to detect the expression alterations of these genes. The results suggested that ULK1 could be steadily regulated by DDX21 (Figure S9A–J and Figure 4G,H). Hence, ULK1 may be a crucial downstream target of DDX21. Interestingly, ULK1 was a sort of serine/threonine kinase, which was a well‐known autophagic initiator, extensively involved in human diseases especially tumours.^27^

Additionally, ChIP‐qPCR assays demonstrated the respective occupancy of DDX21 and YBX1 on ULK1 (Figure 4I,J and Figure S10A–F). Knockdown of YBX1 induced the suppression of ULK1 expression (Figure 4K,L). However, loss of DDX21 had no impacts on YBX1 expression (Figure S11A,B), which meant YBX1 was not directly modulated by DDX21. Intriguingly, when DDX21 was silenced, the binding of YBX1 to ULK1 was remarkably decreased (Figure 4M), suggesting that the recruitment of YBX1 to interact with ULK1 promoter was DDX21‐dependent. Next, to explore whether the recruitment of DDX21 relied on its helicase activity, we designed the helicase‐defective DDX21 plasmids (DDX21^DEV^ and DDX21^SAT^) (Figure S11C,D).^28^ Both WT and mutated DDX21 reversed the diminished affinity of YBX1 to ULK1 promoter caused by DDX21 inhibition (Figure 4N). Mutation of DDX21 helicase activity induced no impacts on interaction, suggesting that the recruitment of YBX1 to ULK1 may be independent of DDX21 enzymatic functions.

In addition, rescue assays with DDX21‐overexpression plus YBX1‐knockdown were performed. As expected, suppression of YBX1 abrogated the promoting effects of DDX21‐overexpression on ULK1 expression (Figure S11E), suggesting the vital role of YBX1 as the mediator in DDX21‐ULK1 axis.

### 3.5 ULK1 strengthened the proliferation abilities of AML cells

**FIGURE 5:**
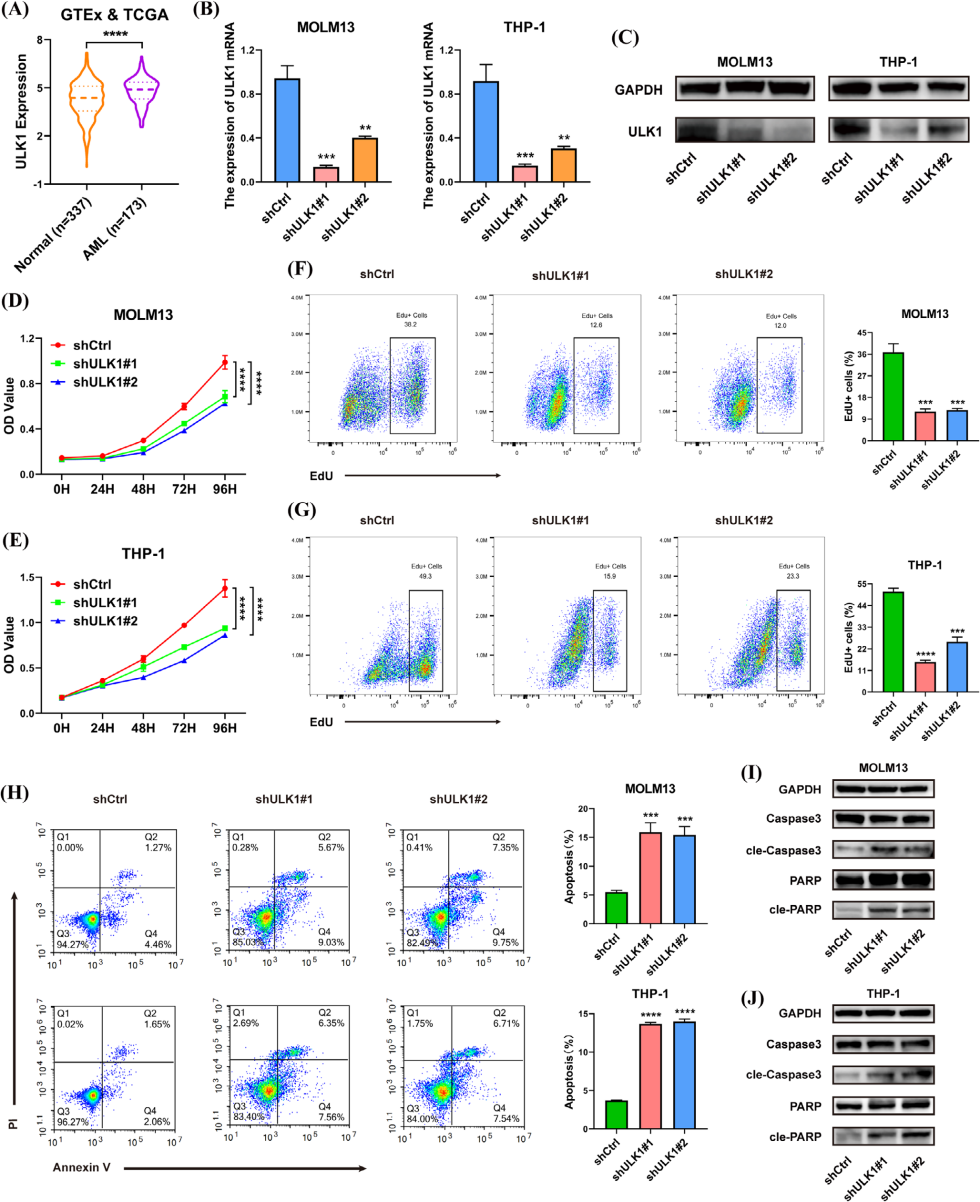
ULK1 was involved in regulating the proliferation and apoptosis of AML cells. (A) The comparison of ULK1 expression between normal (*n* = 337) and AML (*n* = 173) patients was performed based on GTEx and TCGA databases. (B and C) The efficiency of ULK1 silencing was examined in RNA (B) and protein (C) levels. (D and E) CCK‐8 assays were conducted to evaluate the impacts of ULK1 knockdown on cell growth. (F and G) When ULK1 was deficient in MOLM13 (F) and THP‐1 (G) cells, DNA replication capabilities were assessed via EdU assays. (H) The apoptosis of AML cells was measured via flow cytometric analysis after the inhibition of ULK1. (I and J) Western blotting assays detected the changes in expression of apoptosis‐associated proteins (*p < .05, **p < .01, ***p < .001, ****p < .0001).

As mentioned above, ULK1 was regulated by DDX21, whereas its functions in AML remained ambiguous. According to the data from GTEx and TCGA databases, expression of ULK1 was increased in AML patients (Figure 5A), which suggested a poor prognosis (Figure S8E). Then AML cells were transfected with ULK1‐knockdown lentivirus (Figure 5B,C). As a result, the proliferation of AML cells was repressed when ULK1 was deficient (Figure 5D–G). Loss of ULK1 also intensified cell apoptosis (Figure 5H), leading to the enhanced expression of cle‐caspase‐3 and cle‐PARP (Figure 5I,J). In addition, ULK1 knockdown resulted in the cell cycle arrest (Figure S12A). In short, ULK1 is involved in modulating the proliferation of AML cells.

### 3.6 Suppression of ULK1 abrogated the activation effects of DDX21 overexpression

**FIGURE 6:**
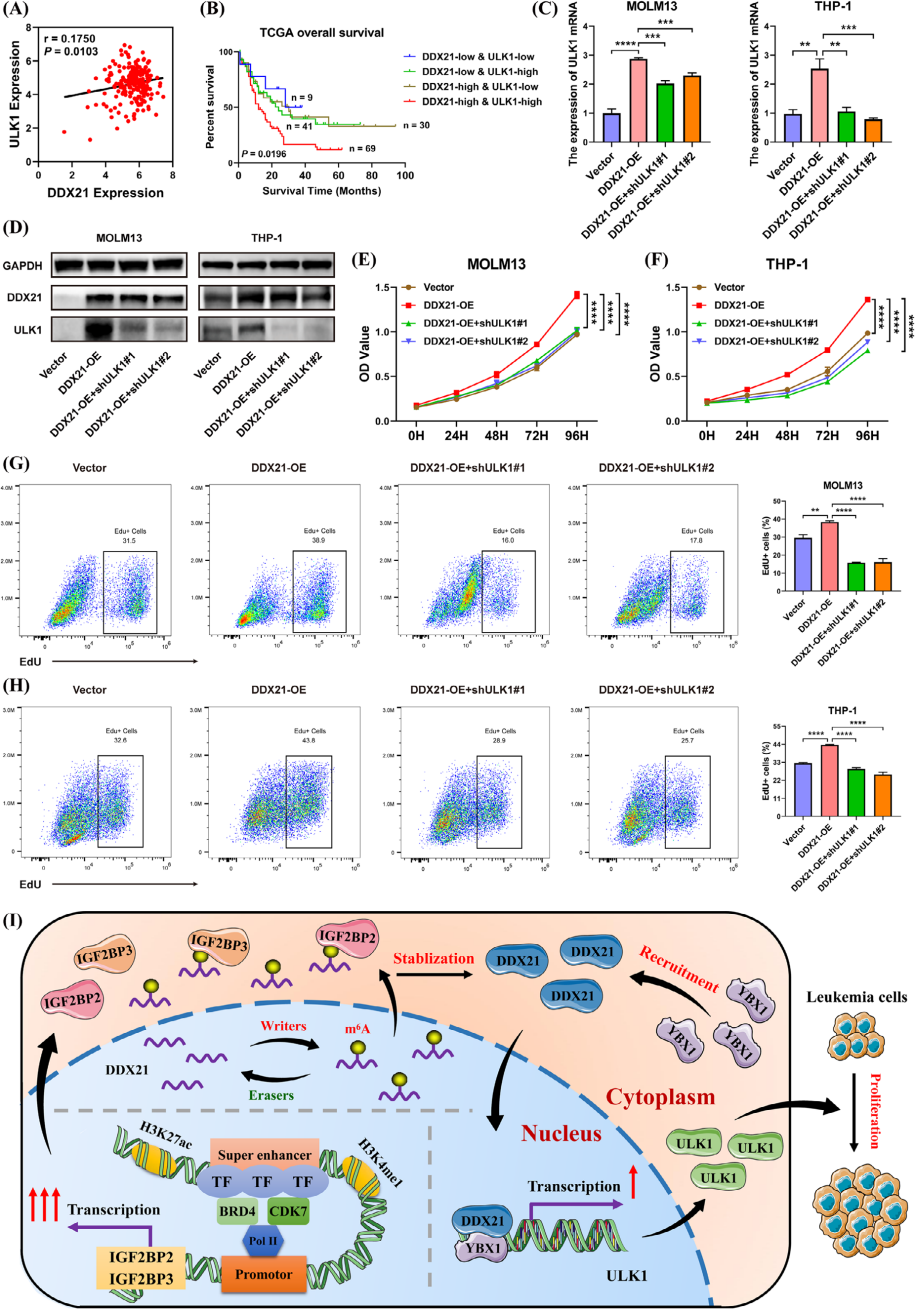
ULK1 suppression reversed the malignant phenotypes induced by DDX21 overexpression. (A) The correlation between DDX21 and ULK1 expression was analysed using TCGA datasets. (B) The survival analysis was conducted in AML patients based on the co‐expression of DDX21 and ULK1 from TCGA datasets. (C and D) The rescue efficiency was validated by RT‐qPCR (C) and Western blotting (D) assays. (E and F) The proliferation of AML cells transfected with empty vector, DDX21‐overexpression and DDX21‐overexpression/ULK1‐knockdown lentivirus was assessed by CCK‐8 assays. (G and H) EdU assays were conducted in the rescue models above (left), and the bar chart shows the DNA duplication abilities of each group (right). (I) A schematic diagram presents the mechanisms that SE‐driven IGF2BP2 and IGF2BP3 trigger the stabilisation of m^6^A‐modified DDX21, which recruits YBX1 to further activate the transcription of ULK1, facilitating the malignancy of AML (*p < .05, **p < .01, ***p < .001, ****p < .0001).

Next, we explored the relationships between ULK1 and DDX21 or YBX1 in AML from TCGA and GEO databases. Either DDX21 or YBX1 was positively correlated with ULK1 in RNA levels (Figure 6A and Figure S12B). In order to identify the impacts of DDX21 and ULK1 on the prognosis of AML patients, survival analysis was conducted based on their co‐expression in TCGA datasets. Unsurprisingly, DDX21^high^ULK1^high^ group had the worst survival (Figure 6B).

In addition, a series of rescue assays were performed to further clarify whether ULK1 was a major functional target of DDX21 in AML cells. DDX21‐overexpression or ULK1‐knockdown plasmids were transfected into AML cells (Figure S12C,D and Figure 6C,D). Overexpression of DDX21 boosted cell growth and DNA duplication, while loss of ULK1 reversed this phenomenon (Figure 6E–H). In conclusion, ULK1 inhibition can rescue the promoting effects induced by DDX21 overexpression, and the DDX21‐ULK1 axis may become the potential target for AML treatment.

## 4 DISCUSSION

Epigenetic dysregulation is extensively involved in diverse physiological and pathological processes, particularly in tumorigenesis. m^6^A RNA methylation and SEs are two important members in epigenetics. m^6^A is generally recognised as a post‐transcriptional modification,^29^ while SEs are predominantly responsible for gene transcription.^12^ Herein, we focus on the link between m^6^A and SEs in AML. Our study identified that the formation of SEs triggered robust activation of IGF2BP2/IGF2BP3 transcription. They then strengthened the stability of m^6^A‐modified DDX21. Furthermore, DDX21 facilitated cell proliferation and inhibited cell apoptosis through recruiting transcription factor YBX1 to cooperatively upregulate ULK1, leading to the progression of AML. In brief, these results unveil the potential functions and mechanisms of SE‐IGF2BP2/IGF2BP3‐DDX21 axis in AML (Figure 6I).

It is noteworthy that m^6^A‐binding proteins act as the vital performers of m^6^A modification, playing essential roles in multiple solid tumours.^8^ Recently, the significance of m^6^A readers in haematological malignancies especially AML has been reported.^30^ Previous studies paid more attention to the individual roles of each m^6^A reader, while we demonstrated the combined effects of IGF2BP2 and IGF2BP3 on m^6^A‐marked transcript. Moreover, upstream regulatory mechanisms of IGF2BPs were scarcely investigated in AML, while we found that IGF2BP2 and IGF2BP3 were driven by SEs, highlighting a novel and potential modulatory pattern of m^6^A‐binding proteins.

Nowadays, accumulating evidence has illuminated the complex crosstalk between m^6^A and other epigenetic regulators. We previously summarised the interplay of m^6^A RNA methylation and DNA methylation, histone modification, chromatin remodelling or non‐coding RNAs, emphasising their indispensable roles in epigenetic reprogramming.^17^ Hence, there may also be a link between m^6^A and SEs. However, merely a few studies confirmed that m^6^A was involved in the modulation of SEs. FTO and METTL14 regulated the stability of SE components and participated in SE‐mediated tumorigenesis.^18^, ^19^ It was still obscure whether SEs had an influence on m^6^A machinery. Encouragingly, our results filled this gap. We revealed that SEs triggered a promoting effect on the expression of IGF2BP2 and IGF2BP3, further impelling the m^6^A‐related malignancy. These findings provide a deeper understanding of the association between SEs and m^6^A.

DEAD‐box RNA helicases, such as DDX41, DDX5 and DDX18, are broadly implicated in AML leukaemogenesis.^31^, ^32^, ^33^ In adult myelodysplastic syndrome/AML patients, germline DDX41 mutations were defined as a significant entity, simultaneously resulting in the susceptibility to AML.^31^, ^32^ Moreover, DDX5 suppression restrained AML progression, while caused no toxicity to normal bone marrow cells.^33^ Until now, the functions of several small molecule inhibitors targeting DEAD‐box RNA helicases have been identified in solid tumours.^34^, ^35^ Our work illustrated the biological effects of DDX21, indicating its potential roles in AML treatment. Nevertheless, no inhibitor targeting DDX21 had been developed so far, which deserved further exploration.

ULK1 is a well‐known autophagic initiator extensively involved in tumorigenesis.^27^ As the terminal effector for SEs‐IGF2BP2/3‐DDX21 axis, further mechanisms of ULK1 merited our attention. AMPK/mTOR/ULK1 pathway was the canonical regulatory axis of autophagy,^36^ and ULK1‐mediated autophagy was tightly associated with drug resistance.^37^ Perhaps DDX21‐triggered ULK1 may induce autophagy to activate drug resistance of AML. Additionally, ULK1 could function beyond autophagy via desensitising cytotoxicity of chemotherapy.^38^ Thus, ULK1 may sustain chromosome instability to dampen curative effects in AML. As high selective inhibitors were available for ULK1,^27^ future studies may focus on the clinical practice of DDX21‐related axis based on ULK1 inhibition.

Indeed, there were some limitations in the current work. First, IGF2BP2, IGF2BP3 and DDX21 were screened out for following investigations mainly based on the data from GEO cohorts. The data of ChIP‐seq, RIP‐seq and MeRIP‐seq utilising our own specimens were insufficient. Second, we just verified the impacts of JQ1 and THZ1 on the expression of IGF2BP2, IGF2BP3 and DDX21. The synergistic effects of SE inhibitors and IGF2BPs inhibitors on AML required further validation. Additionally, perhaps in vivo data such as patient‐derived xenograft (PDX) models could be attempted to extend our findings in the future.

In summary, our findings clarify the potential effects of SE machinery on m^6^A readers, and reveal the underlying functions and mechanisms of IGF2BP2/IGF2BP3‐DDX21 axis in AML, which pave the way to develop more effective therapeutic strategies for AML.

## AUTHOR CONTRIBUTIONS

Yanchun Zhao and Jie Jin designed the project. Yanchun Zhao performed the most of experiments and wrote the paper. Yutong Zhou participated in the establishment of xenograft models. Yu Qian and Wenwen Wei conducted the bioinformatics analyses of AML patients from TCGA, GEO, TARGET and GTEx databases. Xiangjie Lin and Shihui Mao were responsible for statistical analyses. Jie Sun and Jie Jin supervised the study and provided critical suggestions. All authors have read and approved the final manuscript.

## CONFLICT OF INTEREST STATEMENT

The authors declare they have no conflicts of interest.

## ETHICS APPROVAL

All experiments using human specimens and animals in this work were approved by the Ethics Committee of the First Affiliated Hospital of Zhejiang University.

## Supporting information



Supporting Information
