# Supplementary material for: m6A‐dependent upregulation of DDX21 by super‐enhancer‐driven IGF2BP2 and IGF2BP3 facilitates progression of acute myeloid leukaemia
Source: Clin Transl Med. 2024 Apr 4;14(4):e1628. doi: 10.1002/ctm2.1628 (PMC10993053; doi:10.1002/ctm2.1628)
Supplement: Supplementary file 1 — Supporting Information [file CTM2-14-e1628-s001.docx]

**Supporting Information File**

**m^6^A-dependent upregulation of DDX21 by super-enhancer-driven IGF2BP2 and IGF2BP3 facilitates progression of acute myeloid leukemia**

**This file includes:**

Supplementary Plasmid Sequences

Supplementary Figures and corresponding Figure Legends

Supplementary Tables S1-6

**Supplementary Plasmid Sequences**

**The Promoter and Enhancer & Promoter plasmid sequences of IGF2BP2 and IGF2BP3 were showed below, respectively.**

**IGF2BP2-Promoter**

CCACCCTGGCTTTGGAGCTGAACCGGCCGCGCAGCCGGGCGGCGACCATCCGTCCCCCGGCCGCTGACCCCAGCGCTCCGGCCTCGCCGCAGTCCCTGGCCCCTCGGCGTTCGTTTCCCCACCCGCCACCTGGCTTTCGCCGCCGCCGCCCGGGAAGCCCTCGGCACTTCCCAGAATAGAGAGCAGCTGCCAACGTCAGGCTGCCGCCTGCCGCGGGGTTCCGGACACGCGGGGTCTGGGCGGAGAGGACGTGACTCCGCGCTGGCCTTCCGCCTTCCCTCCCCGGTCGGGAAACTGAGGCCCGGGACCGGGAACCACTTCCCCGCGAGCACGGTCCGCGAGGGCCCGGCCAACCCCGGTCCTAGGCGTCCCGACTCCCGCGCGCGATCCTTCGACCACACGCGCCGCTCGGTCACCTCAGAGAGCGTGGCGGGGAGCGGGTCTCAGAGCGAGCGCACGGCCCGGGAACGCGCAGGGCGCCAGCCCCGGCCAAGTCGCTGCGAAAGTTGTTGTCTCCCAGCCTGCCCTCGCCTCTCACCTCCGGGACACACCTTCCCGCTTGCCCTGGCAGCAGCGCACTCACATCCGGTCCACCTTGCGCACACCCCTCTCCGCGCTCACTCGCAGCCCCTCTCCCTCCCACGCCCGCGGCTCCCCGTCGCCCCCTCGCGCGCTCCCACCCGCGCCCGGGGCGCGCCTTCCCCGCCCCGCCGCTCGCCCGGCCTCGGCGCCGCGCCCGCCCGGGGTTCCGGCGCCCGGAGCCCCAGCCCGGGGCCGCGACCCGCTTCCGCCCGCCTGGCGCGGGGCCGGAGCCCGAGCAGCCACTGCCGAGCGCGGTGGGCGCGAGGCGAGGGGCGCAGCGGCGCGGGGCGGGGAGGGGGCGCTCGCTCGCTCCCTCTCGGCTGCGCACACGCCCGTACAAACTCTCACACAGACACACGCGGGGTGCGCTGCCGCCGCCCGCCGCTGCTCCTCCTCCCGCCACCGCCTTGAGAGGGAGAGAGAGGGAGGCAGAGAGAGCGCTTTGTCCGCGCGCCGCCGCCCGGCCCGGGACTCTGCCCCGAGGAGGCAGCCGCGCCGAGTCCCCGCCTCCGCCTCTGCCCCCGGGCGGGCCGGGCCGGCCGCGGTGGGGGGAGCCAGGCTGAGGGTGGGGGTGGGTGGCGGGCGGGCGGAGGGCGGGGAGGGGGGGCGGAGGAGGAG

**IGF2BP2-Enhancer# 1+Promoter**

GCCGGCCCGGCCCGCCCGGGGGCAGAGGCGGAGGCGGGGACTCGGCGCGGCTGCCTCCTCGGGGCAGAGTCCCGGGCCGGGCGGCGGCGCGCGGACAAAGCGCTCTCTCTGCCTCCCTCTCTCTCCCTCTCAAGGCGGTGGCGGGAGGAGGAGCAGCGGCGGGCGGCGGCAGCGCACCCCGCGTGTGTCTGTGTGAGAGTTTGTACGGGCGTGTGCGCAGCCGAGAGGGAGCGAGCGAGCGCCCCCTCCCCGCCCCGCGCCGCTGCGCCCCTCGCCTCGCGCCCACCGCGCTCGGCAGTGGCTGCTCGGGCTCCGGCCCCGCGCCAGGCGGGCGGAAGCGGGTCGCGGCCCCGGGCTGGGGCTCCGGGCGCCGGAACCCCGGGCGGGCGCGGCGCCGAGGCCGGGCGAGCGGCGGGGCGGGGAAGGCGCGCCCCGGGCGCGGGTGGGAGCGCGCGAGGGGGCGACGGGGAGCCGCGGGCGTGGGAGGGAGAGGGGCTGCGAGTGAGCGCGGAGAGGGGTGTGCGCAAGGTGGACCGGATGTGAGTGCGCTGCTGCCAGGGCAAGCGGGAAGGTGTGTCCCGGAGGTGAGAGGCGAGGGCAGGCTGGGAGACAACAACTTTCGCAGCGACTTGGCCGGGGCTGGCGCCCTGCGCGTTCCCGGGCCGTGCGCTCGCTCTGAGACCCGCTCCCCGCCACGCTCTCTGAGGTGACCGAGCGGCGCGTGTGGTCGAAGGATCGCGCGCGGGAGTCGGGACGCCTAGGACCGGGGTTGGCCGGGCCCTCGCGGACCGTGCTCGCGGGGAAGTGGTTCCCGGTCCCGGGCCTCAGTTTCCCGACCGGGGAGGGAAGGCGGAAGGCCAGCGCGGAGTCACGTCCTCTCCGCCCAGACCCCGCGTGTCCGGAACCCCGCGGCAGGCGGCAGCCTGACGTTGGCAGCTGCTCTCTATTCTGGGAAGTGCCGAGGGCTTCCCGGGCGGCGGCGGCGAAAGCCAGGTGGCGGGTGGGGAAACGAACGCCGAGGGGCCAGGGACTGCGGCGAGGCCGGAGCGCTGGGGTCAGCGGCCGGGGGACGGATGGTCGCCGCCCGGCTGCGCGGCCGGTTCAGCTCCAAAGCCAGGGTGGTGGGAGGCGAGGGTGGCTGGAAGCGGAGGAGCGCAGGCTGTGTAGGAGTTCCCCGAGCACTGCCACCCTGGCTTTGGAGCTGAACCGGCCGCGCAGCCGGGCGGCGACCATCCGTCCCCCGGCCGCTGACCCCAGCGCTCCGGCCTCGCCGCAGTCCCTGGCCCCTCGGCGTTCGTTTCCCCACCCGCCACCTGGCTTTCGCCGCCGCCGCCCGGGAAGCCCTCGGCACTTCCCAGAATAGAGAGCAGCTGCCAACGTCAGGCTGCCGCCTGCCGCGGGGTTCCGGACACGCGGGGTCTGGGCGGAGAGGACGTGACTCCGCGCTGGCCTTCCGCCTTCCCTCCCCGGTCGGGAAACTGAGGCCCGGGACCGGGAACCACTTCCCCGCGAGCACGGTCCGCGAGGGCCCGGCCAACCCCGGTCCTAGGCGTCCCGACTCCCGCGCGCGATCCTTCGACCACACGCGCCGCTCGGTCACCTCAGAGAGCGTGGCGGGGAGCGGGTCTCAGAGCGAGCGCACGGCCCGGGAACGCGCAGGGCGCCAGCCCCGGCCAAGTCGCTGCGAAAGTTGTTGTCTCCCAGCCTGCCCTCGCCTCTCACCTCCGGGACACACCTTCCCGCTTGCCCTGGCAGCAGCGCACTCACATCCGGTCCACCTTGCGCACACCCCTCTCCGCGCTCACTCGCAGCCCCTCTCCCTCCCACGCCCGCGGCTCCCCGTCGCCCCCTCGCGCGCTCCCACCCGCGCCCGGGGCGCGCCTTCCCCGCCCCGCCGCTCGCCCGGCCTCGGCGCCGCGCCCGCCCGGGGTTCCGGCGCCCGGAGCCCCAGCCCGGGGCCGCGACCCGCTTCCGCCCGCCTGGCGCGGGGCCGGAGCCCGAGCAGCCACTGCCGAGCGCGGTGGGCGCGAGGCGAGGGGCGCAGCGGCGCGGGGCGGGGAGGGGGCGCTCGCTCGCTCCCTCTCGGCTGCGCACACGCCCGTACAAACTCTCACACAGACACACGCGGGGTGCGCTGCCGCCGCCCGCCGCTGCTCCTCCTCCCGCCACCGCCTTGAGAGGGAGAGAGAGGGAGGCAGAGAGAGCGCTTTGTCCGCGCGCCGCCGCCCGGCCCGGGACTCTGCCCCGAGGAGGCAGCCGCGCCGAGTCCCCGCCTCCGCCTCTGCCCCCGGGCGGGCCGGGCCGGCCGCGGTGGGGGGAGCCAGGCTGAGGGTGGGGGTGGGTGGCGGGCGGGCGGAGGGCGGGGAGGGGGGGCGGAGGAGGAG

**IGF2BP2-Enhancer# 2+Promoter**

CGCGGCAGGCGGCAGCCTGACGTTGGCAGCTGCTCTCTATTCTGGGAAGTGCCGAGGGCTTCCCGGGCGGCGGCGGCGAAAGCCAGGTGGCGGGTGGGGAAACGAACGCCGAGGGGCCAGGGACTGCGGCGAGGCCGGAGCGCTGGGGTCAGCGGCCGGGGGACGGATGGTCGCCGCCCGGCTGCGCGGCCGGTTCAGCTCCAAAGCCAGGGTGGTGGGAGGCGAGGGTGGCTGGAAGCGGAGGAGCGCAGGCTGTGTAGGAGTTCCCCGAGCACTGGGAAGGGCCCGGAGAGGAAAGAGGTCCTTCCAAGCGGAGGAAACAACGTGACCGACGGAAGAGGCAGGGTTGGGATGAGAGCAGGCTGGAGCCGAGGGGCTCGGGCAGGAGCCAGACAGGCTAGAGCGGGCTGCAAGTTCCGGGGCCCGGCTGGAAGCCTGATGGTTAGAGCCTGGTCCCAGCCAGGAAATGAAATTCTACAGGAGGGAGAAGAATGGCGGGATTCAGAGGTGAGGAGGCAGAAGAAGATTCCAAGCTCTGGACCTCTGGGGGCCACTGGGAGAGTTGAAGGTTTGATCGCGGATATGTTGGGTTTTGTGAAGGCGTTGGGGCGCGGGTGCGGGGAGTGTCCTTGGGTGGAGAAGTCACGAATGTAGGAATGGAACACCACCCTGGCTTTGGAGCTGAACCGGCCGCGCAGCCGGGCGGCGACCATCCGTCCCCCGGCCGCTGACCCCAGCGCTCCGGCCTCGCCGCAGTCCCTGGCCCCTCGGCGTTCGTTTCCCCACCCGCCACCTGGCTTTCGCCGCCGCCGCCCGGGAAGCCCTCGGCACTTCCCAGAATAGAGAGCAGCTGCCAACGTCAGGCTGCCGCCTGCCGCGGGGTTCCGGACACGCGGGGTCTGGGCGGAGAGGACGTGACTCCGCGCTGGCCTTCCGCCTTCCCTCCCCGGTCGGGAAACTGAGGCCCGGGACCGGGAACCACTTCCCCGCGAGCACGGTCCGCGAGGGCCCGGCCAACCCCGGTCCTAGGCGTCCCGACTCCCGCGCGCGATCCTTCGACCACACGCGCCGCTCGGTCACCTCAGAGAGCGTGGCGGGGAGCGGGTCTCAGAGCGAGCGCACGGCCCGGGAACGCGCAGGGCGCCAGCCCCGGCCAAGTCGCTGCGAAAGTTGTTGTCTCCCAGCCTGCCCTCGCCTCTCACCTCCGGGACACACCTTCCCGCTTGCCCTGGCAGCAGCGCACTCACATCCGGTCCACCTTGCGCACACCCCTCTCCGCGCTCACTCGCAGCCCCTCTCCCTCCCACGCCCGCGGCTCCCCGTCGCCCCCTCGCGCGCTCCCACCCGCGCCCGGGGCGCGCCTTCCCCGCCCCGCCGCTCGCCCGGCCTCGGCGCCGCGCCCGCCCGGGGTTCCGGCGCCCGGAGCCCCAGCCCGGGGCCGCGACCCGCTTCCGCCCGCCTGGCGCGGGGCCGGAGCCCGAGCAGCCACTGCCGAGCGCGGTGGGCGCGAGGCGAGGGGCGCAGCGGCGCGGGGCGGGGAGGGGGCGCTCGCTCGCTCCCTCTCGGCTGCGCACACGCCCGTACAAACTCTCACACAGACACACGCGGGGTGCGCTGCCGCCGCCCGCCGCTGCTCCTCCTCCCGCCACCGCCTTGAGAGGGAGAGAGAGGGAGGCAGAGAGAGCGCTTTGTCCGCGCGCCGCCGCCCGGCCCGGGACTCTGCCCCGAGGAGGCAGCCGCGCCGAGTCCCCGCCTCCGCCTCTGCCCCCGGGCGGGCCGGGCCGGCCGCGGTGGGGGGAGCCAGGCTGAGGGTGGGGGTGGGTGGCGGGCGGGCGGAGGGCGGGGAGGGGGGGCGGAGGAGGAG

**IGF2BP3-Promoter**

CTTAAATAAAAGTACCAGCCTACTCCACTCCTCCCCCTTTAAAAACTACAGGAAGAGAGGGTTTCCCTTTACCGACTTTTCCCACTTTGACTTCTCTCCTCCAGCGGCCCAGGCCCCCGACGACCCCAGCCCCTAGCCAGAAACCTCCGCCCCTGCACTCCCCTCCCCCACCAGTGGCCAAGCCCGCCCCCGGCCTCATTTATCCTGGAGCCTTTTCTGAATCGCGCAACCAATGGCAAACGCGAAGCCGAGAGCCACCGCGAGCGCAGGCGGAGGCGGAGGAGGTGTCGCCCAGACACCCGCCCAGGACGCTGCGGGATCCCATCCCCCACCACTCCGGGGCCGGGCCGCCCCCACTCGCGCGGTGCCCAGGGCCCGAGAAGGGCGCACGGGGTGGGGCGCGTCGCCCGCCTGTCTTGCCCCCACCCCCTGCCCCCCTCACCCACTCCCCCACCCCTCCTCGCAGCATTTCCGTCCATGCCAGCCTTAGAAACGTGCCTGCGGCTTTGCTGGGGTTACACTTCTAATGCCATTAAAGCCACCAGGCGAGTTCGGGATTGTGACTCTGGTTTTGTTGTTAATAACCTATTTTGAAAAATCGAATGATTAAAACTACGGTAACTAGCGCTGAGGAACTGCCTGTTAGTACCTCTGCCCATTTCCTCATTAAAAAAAAAAAAATCAATATTCCAGCCCTCCCTCCCAGGATCCGCCCAGACCAGATAACCCTTTACAAACTCGAGTTGTGACCGTCAGCGCCCCGCTTGGTGCCGGCTGCGGTTCCTTTAGCATTTTCCTTCTTCCTCCTCCCCCTTCTCAGAGTCCCTCTCCCAATCTCGTTTCCCCTCCCCCTCTTCCTCTCGCTCGCTTTTTTCTCCGAAACCCACTCTAATTGAGGCTCTGGGATTCCTCACGTGAGACGTTGATTTGTAGTTTGAACACGTGGCTCATTCAAAAAGCCGGACACTCGGAGCGGATTTTTTCCGCCTCCTGCGCCTTCCTCCCTCCTCCTCCTCCTCCTCTCCCCTCCCTCCTGCCAGTCACCCTTCTGGGTTTTTTATGGGGAGGCGGCGCCTGCCTCACGTAGTGTGATATTTTCACAGTCCGCTGGCCGAAGCCAAAGGGGTTGGGAAGGAAGAGACAAAAAGTAGTTTTTTCCCCTCCTCCTCCTCCTTTCTCTTGCTAATCCCGCCTCCTCCT

**IGF2BP3-Enhancer# 1+Promoter**

GCGCGAGTGGGGGCGGCCCGGCCCCGGAGTGGTGGGGGATGGGATCCCGCAGCGTCCTGGGCGGGTGTCTGGGCGACACCTCCTCCGCCTCCGCCTGCGCTCGCGGTGGCTCTCGGCTTCGCGTTTGCCATTGGTTGCGCGATTCAGAAAAGGCTCCAGGATAAATGAGGCCGGGGGCGGGCTTGGCCACTGGTGGGGGAGGGGAGTGCAGGGGCGGAGGTTTCTGGCTAGGGGCTGGGGTCGTCGGGGGCCTGGGCCGCTGGAGGAGAGAAGTCAAAGTGGGAAAAGTCGGTAAAGGGAAACCCTCTCTTCCTGTAGTTTTTAAAGGGGGAGGAGTGGAGTAGGCTGGTACTTTTATTTAAGTAATTTTAGTTGGAGAACTTGAGACTTAAAACTGGAAGACCGTCGTGCATTACCCCTAGGTGGTATTCGCATTTGAAAAATTGTTCGAGTCTCTTTCTGCCTCTTTTCTGCCTGGATTTGGCCATGAGAGCCATACATAATTTTCTCTATATGTAATGAGTACAATGGTGGTTGTCGGGGGAGGATTTGGGCCCGGTGGAGCCTTGTGGGCCAACAAAATGTGAACTGGAGACGAGCAATACGGAAACGAAGGCAGTGCTGGGGTTCTAGTGGAATCTCCAACTGTCGTTTAAAGATTGTGTACTCCACTCAAAGGTTTCATTTGCACCAGATACTTGTGTGTTTTTAACAGGTATATTTTTAACAGGTATTCTATTGAAAAGATAGAAAATTCTAATTTTTTAAGTGAAAGAAATTCTATATAACATGAAAATATAGACAAGAATCTTTAATGACTTGGTACTCTTACTGGTAGAAAAAAGGTATCCTGAATAGAGGAAATATTATGATTTACACTGGAAACATCTTCGGTAATTGATACAGCCTTGCTTCTCTGTCAAAACCGTTGTTAGCATTTTAAAATGTGTATTTTTCATTTAAAAGATGAATGGTAGACTTAAACATGGGAATTGTTGCATGATATAAAGAGTATTTACATTTTGTCTTTTAAAGGGAACTTACTTTGTAATTTTGTGTTAAGGAACCGGAAGGTGTCAGATCATCCATTTTAAATGCAACTGTTCTTTCTTACAGGTTTTTCTTTCCTGCCAAGCGAATCCAACATTTTAAACGTAAACACACTTTCACAATAATTTCATTCACGTTTAATTTAATAAAATGTTGTTACTTTATAAAATAGCTTAAATAAAAGTACCAGCCTACTCCACTCCTCCCCCTTTAAAAACTACAGGAAGAGAGGGTTTCCCTTTACCGACTTTTCCCACTTTGACTTCTCTCCTCCAGCGGCCCAGGCCCCCGACGACCCCAGCCCCTAGCCAGAAACCTCCGCCCCTGCACTCCCCTCCCCCACCAGTGGCCAAGCCCGCCCCCGGCCTCATTTATCCTGGAGCCTTTTCTGAATCGCGCAACCAATGGCAAACGCGAAGCCGAGAGCCACCGCGAGCGCAGGCGGAGGCGGAGGAGGTGTCGCCCAGACACCCGCCCAGGACGCTGCGGGATCCCATCCCCCACCACTCCGGGGCCGGGCCGCCCCCACTCGCGCGGTGCCCAGGGCCCGAGAAGGGCGCACGGGGTGGGGCGCGTCGCCCGCCTGTCTTGCCCCCACCCCCTGCCCCCCTCACCCACTCCCCCACCCCTCCTCGCAGCATTTCCGTCCATGCCAGCCTTAGAAACGTGCCTGCGGCTTTGCTGGGGTTACACTTCTAATGCCATTAAAGCCACCAGGCGAGTTCGGGATTGTGACTCTGGTTTTGTTGTTAATAACCTATTTTGAAAAATCGAATGATTAAAACTACGGTAACTAGCGCTGAGGAACTGCCTGTTAGTACCTCTGCCCATTTCCTCATTAAAAAAAAAAAAATCAATATTCCAGCCCTCCCTCCCAGGATCCGCCCAGACCAGATAACCCTTTACAAACTCGAGTTGTGACCGTCAGCGCCCCGCTTGGTGCCGGCTGCGGTTCCTTTAGCATTTTCCTTCTTCCTCCTCCCCCTTCTCAGAGTCCCTCTCCCAATCTCGTTTCCCCTCCCCCTCTTCCTCTCGCTCGCTTTTTTCTCCGAAACCCACTCTAATTGAGGCTCTGGGATTCCTCACGTGAGACGTTGATTTGTAGTTTGAACACGTGGCTCATTCAAAAAGCCGGACACTCGGAGCGGATTTTTTCCGCCTCCTGCGCCTTCCTCCCTCCTCCTCCTCCTCCTCTCCCCTCCCTCCTGCCAGTCACCCTTCTGGGTTTTTTATGGGGAGGCGGCGCCTGCCTCACGTAGTGTGATATTTTCACAGTCCGCTGGCCGAAGCCAAAGGGGTTGGGAAGGAAGAGACAAAAAGTAGTTTTTTCCCCTCCTCCTCCTCCTTTCTCTTGCTAATCCCGCCTCCTCCT

**IGF2BP3-Enhancer# 2+Promoter**

GCTGCATCTCCTCCTTATCTGCTGAACTGAGGACTGTACAAATGTTTCTTGTGGGTCAGTGTAATATTAAAGAATAGCTATGTTTAGAAGGCCAGTTACCGTTGCTGTACCCACACGTCAGGGGCAAAGGCTGAGTGGGCAGATGGGGGGCTGCCACCCCTCCTCACGCCCGGCTTGGGACCAGCCTGGAGTCTCAAGCCGAAACCCCTCACGCGGGTGGGCACACAATGCCCGGCGTGTCGCCTGGGCCTCCTCGGAGGTGCTTTAACAGGCTCTTGCGCTTTTGAACGGCCCTCCTGGACTCTGATCTGTTTAAACTAGAGGCGTTAATAAGTGATAACCTGGATCTGCCCTCCTTGGAAGCTGCCTGCCTATATTTTATTGTTTAAAATAAATAAAAATGGTGCCCCGGGAGAGAAGATGGGGTTGTTTCCTTTTTCCTCTGGCTCTGCCCCGAGAAAGAGGGCGGGATCCTCACGGCTCACAGTGGGGAGGGTCTTTCGTCGGCCGCCGGCTCGCCTCGGAGGGCCTGGACCACTGGACCGCCCTTCCCCGGGGCCCTGCCGCGGGACCGCGGGCTTCTGGCTCCTCCTGGGGCTGCGTCCGTATGCGCGGAGCGTGTGGCCGGGCCGTCGCCGCGCCACCCCACCTGAGTCCGCCGGCCAGCGCGGGGACGCACCGGGCAGCGTGTGTTTGGCGACCCTCCCGCACCTCTGGTCTCAGTTGCGTGTGTGCACGAGGGGTTCCATAGGGCCCAGGGATGCTTGGTACCCACGGGGGAGAATCCCTCGCCGAACCCTGCGGGTCTGCGGGGCGGGCCGCGAGACTGGCGCGCAAAAGCGGCTCCAAGGCGGGGCTCCCGCGCTCCCCGGGGCCGGCTTGCCGAGTCCAAGTTGAGCAACCGGCGTCGAGAGAGACACCGCCCCTGCTGCGGGCGGGGGCCTCTCCTCGCTTCCGATTGGCTGACGGGGGGAACCTATCGCCGTCGGCCGCCTCCGCCAGAGCGGTTTGCTGGTTTTCATTCATTGGCCCCGGAGCCGCCCCTGGATTTCCATCTTTTGTGGCGCGAAAATAACCCTTTGCTCCCTCGTTGGTTTTGTTGAGGTTGAGGGGTGGGACTGTGTTCCCCTCTGCTCGCTCTCGTTTTTCCTGCCCTTTAACAGCTCGCCCCCAGCCCCAACTTAAATAAAAGTACCAGCCTACTCCACTCCTCCCCCTTTAAAAACTACAGGAAGAGAGGGTTTCCCTTTACCGACTTTTCCCACTTTGACTTCTCTCCTCCAGCGGCCCAGGCCCCCGACGACCCCAGCCCCTAGCCAGAAACCTCCGCCCCTGCACTCCCCTCCCCCACCAGTGGCCAAGCCCGCCCCCGGCCTCATTTATCCTGGAGCCTTTTCTGAATCGCGCAACCAATGGCAAACGCGAAGCCGAGAGCCACCGCGAGCGCAGGCGGAGGCGGAGGAGGTGTCGCCCAGACACCCGCCCAGGACGCTGCGGGATCCCATCCCCCACCACTCCGGGGCCGGGCCGCCCCCACTCGCGCGGTGCCCAGGGCCCGAGAAGGGCGCACGGGGTGGGGCGCGTCGCCCGCCTGTCTTGCCCCCACCCCCTGCCCCCCTCACCCACTCCCCCACCCCTCCTCGCAGCATTTCCGTCCATGCCAGCCTTAGAAACGTGCCTGCGGCTTTGCTGGGGTTACACTTCTAATGCCATTAAAGCCACCAGGCGAGTTCGGGATTGTGACTCTGGTTTTGTTGTTAATAACCTATTTTGAAAAATCGAATGATTAAAACTACGGTAACTAGCGCTGAGGAACTGCCTGTTAGTACCTCTGCCCATTTCCTCATTAAAAAAAAAAAAATCAATATTCCAGCCCTCCCTCCCAGGATCCGCCCAGACCAGATAACCCTTTACAAACTCGAGTTGTGACCGTCAGCGCCCCGCTTGGTGCCGGCTGCGGTTCCTTTAGCATTTTCCTTCTTCCTCCTCCCCCTTCTCAGAGTCCCTCTCCCAATCTCGTTTCCCCTCCCCCTCTTCCTCTCGCTCGCTTTTTTCTCCGAAACCCACTCTAATTGAGGCTCTGGGATTCCTCACGTGAGACGTTGATTTGTAGTTTGAACACGTGGCTCATTCAAAAAGCCGGACACTCGGAGCGGATTTTTTCCGCCTCCTGCGCCTTCCTCCCTCCTCCTCCTCCTCCTCTCCCCTCCCTCCTGCCAGTCACCCTTCTGGGTTTTTTATGGGGAGGCGGCGCCTGCCTCACGTAGTGTGATATTTTCACAGTCCGCTGGCCGAAGCCAAAGGGGTTGGGAAGGAAGAGACAAAAAGTAGTTTTTTCCCCTCCTCCTCCTCCTTTCTCTTGCTAATCCCGCCTCCTCCT

**The wild-type (WT) and truncation mutant (M1-M4) plasmid sequences of IGF2BP2 and IGF2BP3 were showed below, respectively.**

**IGF2BP2-WT**

ATGATGAACAAGCTTTACATCGGGAACCTGAGCCCCGCCGTCACCGCCGACGACCTCCGGCAGCTCTTTGGGGACAGGAAGCTGCCCCTGGCGGGACAGGTCCTGCTGAAGTCCGGCTACGCCTTCGTGGACTACCCCGACCAGAACTGGGCCATCCGCGCCATCGAGACCCTCTCGGGTAAAGTGGAATTGCATGGGAAAATCATGGAAGTTGATTACTCAGTCTCTAAAAAGCTAAGGAGCAGGAAAATTCAGATTCGAAACATCCCTCCTCACCTGCAGTGGGAGGTGTTGGATGGACTTTTGGCTCAATATGGGACAGTGGAGAATGTGGAACAAGTCAACACAGACACAGAAACCGCCGTTGTCAACGTCACATATGCAACAAGAGAAGAAGCAAAAATAGCCATGGAGAAGCTAAGCGGGCATCAGTTTGAGAACTACTCCTTCAAGATTTCCTACATCCCGGATGAAGAGGTGAGCTCCCCTTCGCCCCCTCAGCGAGCCCAGCGTGGGGACCACTCTTCCCGGGAGCAAGGCCACGCCCCTGGGGGCACTTCTCAGGCCAGACAGATTGATTTCCCGCTGCGGATCCTGGTCCCCACCCAGTTTGTTGGTGCCATCATCGGAAAGGAGGGCTTGACCATAAAGAACATCACTAAGCAGACCCAGTCCCGGGTAGATATCCATAGAAAAGAGAACTCTGGAGCTGCAGAGAAGCCTGTCACCATCCATGCCACCCCAGAGGGGACTTCTGAAGCATGCCGCATGATTCTTGAAATCATGCAGAAAGAGGCAGATGAGACCAAACTAGCCGAAGAGATTCCTCTGAAAATCTTGGCACACAATGGCTTGGTTGGAAGACTGATTGGAAAAGAAGGCAGAAATTTGAAGAAAATTGAACATGAAACAGGGACCAAGATAACAATCTCATCTTTGCAGGATTTGAGCATATACAACCCGGAAAGAACCATCACTGTGAAGGGCACAGTTGAGGCCTGTGCCAGTGCTGAGATAGAGATTATGAAGAAGCTGCGTGAGGCCTTTGAAAATGATATGCTGGCTGTTAACCAACAAGCCAATCTGATCCCAGGGTTGAACCTCAGCGCACTTGGCATCTTTTCAACAGGACTGTCCGTGCTATCTCCACCAGCAGGGCCCCGCGGAGCTCCCCCCGCTGCCCCCTACCACCCCTTCACTACCCACTCCGGATACTTCTCCAGCCTGTACCCCCATCACCAGTTTGGCCCGTTCCCGCATCATCACTCTTATCCAGAGCAGGAGATTGTGAATCTCTTCATCCCAACCCAGGCTGTGGGCGCCATCATCGGGAAGAAGGGGGCACACATCAAACAGCTGGCGAGATTCGCCGGAGCCTCTATCAAGATTGCCCCTGCGGAAGGCCCAGACGTCAGCGAAAGGATGGTCATCATCACCGGGCCACCGGAAGCCCAGTTCAAGGCCCAGGGACGGATCTTTGGGAAACTGAAAGAGGAAAACTTCTTTAACCCCAAAGAAGAAGTGAAGCTGGAAGCGCATATCAGAGTGCCCTCTTCCACAGCTGGCCGGGTGATTGGCAAAGGTGGCAAGACCGTGAACGAACTGCAGAACTTAACCAGTGCAGAAGTCATCGTGCCTCGTGACCAAACGCCAGATGAAAATGAGGAAGTGATCGTCAGAATTATCGGGCACTTCTTTGCTAGCCAGACTGCACAGCGCAAGATCAGGGAAATTGTACAACAGGTGAAGCAGCAGGAGCAGAAATACCCTCAGGGAGTCGCCTCACAGCGCAGCAAG

**IGF2BP2-M1 (Del KH1-2)**

ATGATGAACAAGCTTTACATCGGGAACCTGAGCCCCGCCGTCACCGCCGACGACCTCCGGCAGCTCTTTGGGGACAGGAAGCTGCCCCTGGCGGGACAGGTCCTGCTGAAGTCCGGCTACGCCTTCGTGGACTACCCCGACCAGAACTGGGCCATCCGCGCCATCGAGACCCTCTCGGGTAAAGTGGAATTGCATGGGAAAATCATGGAAGTTGATTACTCAGTCTCTAAAAAGCTAAGGAGCAGGAAAATTCAGATTCGAAACATCCCTCCTCACCTGCAGTGGGAGGTGTTGGATGGACTTTTGGCTCAATATGGGACAGTGGAGAATGTGGAACAAGTCAACACAGACACAGAAACCGCCGTTGTCAACGTCACATATGCAACAAGAGAAGAAGCAAAAATAGCCATGGAGAAGCTAAGCGGGCATCAGTTTGAGAACTACTCCTTCAAGATTTCCTACATCCCGGATGAAGAGGTGAGCTCCCCTTCGCCCCCTCAGCGAGCCCAGCGTGGGGACCACTCTTCCCGGGAGCAAGGCCACGCCCCTGGGGGCACTTCTCAGGCCAGACAGATTCTTGAAATCATGCAGAAAGAGGCAGATGAGACCAAACTAGCCGAAATGAAGAAGCTGCGTGAGGCCTTTGAAAATGATATGCTGGCTGTTAACCAACAAGCCAATCTGATCCCAGGGTTGAACCTCAGCGCACTTGGCATCTTTTCAACAGGACTGTCCGTGCTATCTCCACCAGCAGGGCCCCGCGGAGCTCCCCCCGCTGCCCCCTACCACCCCTTCACTACCCACTCCGGATACTTCTCCAGCCTGTACCCCCATCACCAGTTTGGCCCGTTCCCGCATCATCACTCTTATCCAGAGCAGGAGATTGTGAATCTCTTCATCCCAACCCAGGCTGTGGGCGCCATCATCGGGAAGAAGGGGGCACACATCAAACAGCTGGCGAGATTCGCCGGAGCCTCTATCAAGATTGCCCCTGCGGAAGGCCCAGACGTCAGCGAAAGGATGGTCATCATCACCGGGCCACCGGAAGCCCAGTTCAAGGCCCAGGGACGGATCTTTGGGAAACTGAAAGAGGAAAACTTCTTTAACCCCAAAGAAGAAGTGAAGCTGGAAGCGCATATCAGAGTGCCCTCTTCCACAGCTGGCCGGGTGATTGGCAAAGGTGGCAAGACCGTGAACGAACTGCAGAACTTAACCAGTGCAGAAGTCATCGTGCCTCGTGACCAAACGCCAGATGAAAATGAGGAAGTGATCGTCAGAATTATCGGGCACTTCTTTGCTAGCCAGACTGCACAGCGCAAGATCAGGGAAATTGTACAACAGGTGAAGCAGCAGGAGCAGAAATACCCTCAGGGAGTCGCCTCACAGCGCAGCAAG

**IGF2BP2-M2 (Del KH3-4)**

ATGATGAACAAGCTTTACATCGGGAACCTGAGCCCCGCCGTCACCGCCGACGACCTCCGGCAGCTCTTTGGGGACAGGAAGCTGCCCCTGGCGGGACAGGTCCTGCTGAAGTCCGGCTACGCCTTCGTGGACTACCCCGACCAGAACTGGGCCATCCGCGCCATCGAGACCCTCTCGGGTAAAGTGGAATTGCATGGGAAAATCATGGAAGTTGATTACTCAGTCTCTAAAAAGCTAAGGAGCAGGAAAATTCAGATTCGAAACATCCCTCCTCACCTGCAGTGGGAGGTGTTGGATGGACTTTTGGCTCAATATGGGACAGTGGAGAATGTGGAACAAGTCAACACAGACACAGAAACCGCCGTTGTCAACGTCACATATGCAACAAGAGAAGAAGCAAAAATAGCCATGGAGAAGCTAAGCGGGCATCAGTTTGAGAACTACTCCTTCAAGATTTCCTACATCCCGGATGAAGAGGTGAGCTCCCCTTCGCCCCCTCAGCGAGCCCAGCGTGGGGACCACTCTTCCCGGGAGCAAGGCCACGCCCCTGGGGGCACTTCTCAGGCCAGACAGATTGATTTCCCGCTGCGGATCCTGGTCCCCACCCAGTTTGTTGGTGCCATCATCGGAAAGGAGGGCTTGACCATAAAGAACATCACTAAGCAGACCCAGTCCCGGGTAGATATCCATAGAAAAGAGAACTCTGGAGCTGCAGAGAAGCCTGTCACCATCCATGCCACCCCAGAGGGGACTTCTGAAGCATGCCGCATGATTCTTGAAATCATGCAGAAAGAGGCAGATGAGACCAAACTAGCCGAAGAGATTCCTCTGAAAATCTTGGCACACAATGGCTTGGTTGGAAGACTGATTGGAAAAGAAGGCAGAAATTTGAAGAAAATTGAACATGAAACAGGGACCAAGATAACAATCTCATCTTTGCAGGATTTGAGCATATACAACCCGGAAAGAACCATCACTGTGAAGGGCACAGTTGAGGCCTGTGCCAGTGCTGAGATAGAGATTATGAAGAAGCTGCGTGAGGCCTTTGAAAATGATATGCTGGCTGTTAACCAACAAGCCAATCTGATCCCAGGGTTGAACCTCAGCGCACTTGGCATCTTTTCAACAGGACTGTCCGTGCTATCTCCACCAGCAGGGCCCCGCGGAGCTCCCCCCGCTGCCCCCTACCACCCCTTCACTACCCACTCCGGATACTTCTCCAGCCTGTACCCCCATCACCAGTTTGGCCCGTTCCCGCATCATCACTCTTATCCAGAGTTTGGGAAACTGAAAGAGGAAAACTTCTTTAACCCCAAAGAAGAAGTGAGGGAAATTGTACAACAGGTGAAGCAGCAGGAGCAGAAATACCCTCAGGGAGTCGCCTCACAGCGCAGCAAG

**IGF2BP2-M3 (Del RRM1-2)**

ATGATGAAAAAGCTAAGGAGCGAAGAGGTGAGCTCCCCTTCGCCCCCTCAGCGAGCCCAGCGTGGGGACCACTCTTCCCGGGAGCAAGGCCACGCCCCTGGGGGCACTTCTCAGGCCAGACAGATTGATTTCCCGCTGCGGATCCTGGTCCCCACCCAGTTTGTTGGTGCCATCATCGGAAAGGAGGGCTTGACCATAAAGAACATCACTAAGCAGACCCAGTCCCGGGTAGATATCCATAGAAAAGAGAACTCTGGAGCTGCAGAGAAGCCTGTCACCATCCATGCCACCCCAGAGGGGACTTCTGAAGCATGCCGCATGATTCTTGAAATCATGCAGAAAGAGGCAGATGAGACCAAACTAGCCGAAGAGATTCCTCTGAAAATCTTGGCACACAATGGCTTGGTTGGAAGACTGATTGGAAAAGAAGGCAGAAATTTGAAGAAAATTGAACATGAAACAGGGACCAAGATAACAATCTCATCTTTGCAGGATTTGAGCATATACAACCCGGAAAGAACCATCACTGTGAAGGGCACAGTTGAGGCCTGTGCCAGTGCTGAGATAGAGATTATGAAGAAGCTGCGTGAGGCCTTTGAAAATGATATGCTGGCTGTTAACCAACAAGCCAATCTGATCCCAGGGTTGAACCTCAGCGCACTTGGCATCTTTTCAACAGGACTGTCCGTGCTATCTCCACCAGCAGGGCCCCGCGGAGCTCCCCCCGCTGCCCCCTACCACCCCTTCACTACCCACTCCGGATACTTCTCCAGCCTGTACCCCCATCACCAGTTTGGCCCGTTCCCGCATCATCACTCTTATCCAGAGCAGGAGATTGTGAATCTCTTCATCCCAACCCAGGCTGTGGGCGCCATCATCGGGAAGAAGGGGGCACACATCAAACAGCTGGCGAGATTCGCCGGAGCCTCTATCAAGATTGCCCCTGCGGAAGGCCCAGACGTCAGCGAAAGGATGGTCATCATCACCGGGCCACCGGAAGCCCAGTTCAAGGCCCAGGGACGGATCTTTGGGAAACTGAAAGAGGAAAACTTCTTTAACCCCAAAGAAGAAGTGAAGCTGGAAGCGCATATCAGAGTGCCCTCTTCCACAGCTGGCCGGGTGATTGGCAAAGGTGGCAAGACCGTGAACGAACTGCAGAACTTAACCAGTGCAGAAGTCATCGTGCCTCGTGACCAAACGCCAGATGAAAATGAGGAAGTGATCGTCAGAATTATCGGGCACTTCTTTGCTAGCCAGACTGCACAGCGCAAGATCAGGGAAATTGTACAACAGGTGAAGCAGCAGGAGCAGAAATACCCTCAGGGAGTCGCCTCACAGCGCAGCAAG

**IGF2BP2-M4 (Del KH1-4)**

ATGATGAACAAGCTTTACATCGGGAACCTGAGCCCCGCCGTCACCGCCGACGACCTCCGGCAGCTCTTTGGGGACAGGAAGCTGCCCCTGGCGGGACAGGTCCTGCTGAAGTCCGGCTACGCCTTCGTGGACTACCCCGACCAGAACTGGGCCATCCGCGCCATCGAGACCCTCTCGGGTAAAGTGGAATTGCATGGGAAAATCATGGAAGTTGATTACTCAGTCTCTAAAAAGCTAAGGAGCAGGAAAATTCAGATTCGAAACATCCCTCCTCACCTGCAGTGGGAGGTGTTGGATGGACTTTTGGCTCAATATGGGACAGTGGAGAATGTGGAACAAGTCAACACAGACACAGAAACCGCCGTTGTCAACGTCACATATGCAACAAGAGAAGAAGCAAAAATAGCCATGGAGAAGCTAAGCGGGCATCAGTTTGAGAACTACTCCTTCAAGATTTCCTACATCCCGGATGAAGAGGTGAGCTCCCCTTCGCCCCCTCAGCGAGCCCAGCGTGGGGACCACTCTTCCCGGGAGCAAGGCCACGCCCCTGGGGGCACTTCTCAGGCCAGACAGATTCTTGAAATCATGCAGAAAGAGGCAGATGAGACCAAACTAGCCGAAATGAAGAAGCTGCGTGAGGCCTTTGAAAATGATATGCTGGCTGTTAACCAACAAGCCAATCTGATCCCAGGGTTGAACCTCAGCGCACTTGGCATCTTTTCAACAGGACTGTCCGTGCTATCTCCACCAGCAGGGCCCCGCGGAGCTCCCCCCGCTGCCCCCTACCACCCCTTCACTACCCACTCCGGATACTTCTCCAGCCTGTACCCCCATCACCAGTTTGGCCCGTTCCCGCATCATCACTCTTATCCAGAGTTTGGGAAACTGAAAGAGGAAAACTTCTTTAACCCCAAAGAAGAAGTGAGGGAAATTGTACAACAGGTGAAGCAGCAGGAGCAGAAATACCCTCAGGGAGTCGCCTCACAGCGCAGCAAG

**IGF2BP3-WT**

ATGAACAAACTGTATATCGGAAACCTCAGCGAGAACGCCGCCCCCTCGGACCTAGAAAGTATCTTCAAGGACGCCAAGATCCCGGTGTCGGGACCCTTCCTGGTGAAGACTGGCTACGCGTTCGTGGACTGCCCGGACGAGAGCTGGGCCCTCAAGGCCATCGAGGCGCTTTCAGGTAAAATAGAACTGCACGGGAAACCCATAGAAGTTGAGCACTCGGTCCCAAAAAGGCAAAGGATTCGGAAACTTCAGATACGAAATATCCCGCCTCATTTACAGTGGGAGGTGCTGGATAGTTTACTAGTCCAGTATGGAGTGGTGGAGAGCTGTGAGCAAGTGAACACTGACTCGGAAACTGCAGTTGTAAATGTAACCTATTCCAGTAAGGACCAAGCTAGACAAGCACTAGACAAACTGAATGGATTTCAGTTAGAGAATTTCACCTTGAAAGTAGCCTATATCCCTGATGAAATGGCCGCCCAGCAAAACCCCTTGCAGCAGCCCCGAGGTCGCCGGGGGCTTGGGCAGAGGGGCTCCTCAAGGCAGGGGTCTCCAGGATCCGTATCCAAGCAGAAACCATGTGATTTGCCTCTGCGCCTGCTGGTTCCCACCCAATTTGTTGGAGCCATCATAGGAAAAGAAGGTGCCACCATTCGGAACATCACCAAACAGACCCAGTCTAAAATCGATGTCCACCGTAAAGAAAATGCGGGGGCTGCTGAGAAGTCGATTACTATCCTCTCTACTCCTGAAGGCACCTCTGCGGCTTGTAAGTCTATTCTGGAGATTATGCATAAGGAAGCTCAAGATATAAAATTCACAGAAGAGATCCCCTTGAAGATTTTAGCTCATAATAACTTTGTTGGACGTCTTATTGGTAAAGAAGGAAGAAATCTTAAAAAAATTGAGCAAGACACAGACACTAAAATCACGATATCTCCATTGCAGGAATTGACGCTGTATAATCCAGAACGCACTATTACAGTTAAAGGCAATGTTGAGACATGTGCCAAAGCTGAGGAGGAGATCATGAAGAAAATCAGGGAGTCTTATGAAAATGATATTGCTTCTATGAATCTTCAAGCACATTTAATTCCTGGATTAAATCTGAACGCCTTGGGTCTGTTCCCACCCACTTCAGGGATGCCACCTCCCACCTCAGGGCCCCCTTCAGCCATGACTCCTCCCTACCCGCAGTTTGAGCAATCAGAAACGGAGACTGTTCATCTGTTTATCCCAGCTCTATCAGTCGGTGCCATCATCGGCAAGCAGGGCCAGCACATCAAGCAGCTTTCTCGCTTTGCTGGAGCTTCAATTAAGATTGCTCCAGCGGAAGCACCAGATGCTAAAGTGAGGATGGTGATTATCACTGGACCACCAGAGGCTCAGTTCAAGGCTCAGGGAAGAATTTATGGAAAAATTAAAGAAGAAAACTTTGTTAGTCCTAAAGAAGAGGTGAAACTTGAAGCTCATATCAGAGTGCCATCCTTTGCTGCTGGCAGAGTTATTGGAAAAGGAGGCAAAACGGTGAATGAACTTCAGAATTTGTCAAGTGCAGAAGTTGTTGTCCCTCGTGACCAGACACCTGATGAGAATGACCAAGTGGTTGTCAAAATAACTGGTCACTTCTATGCTTGCCAGGTTGCCCAGAGAAAAATTCAGGAAATTCTGACTCAGGTAAAGCAGCACCAACAACAGAAGGCTCTGCAAAGTGGACCACCTCAGTCAAGACGGAAG

**IGF2BP3-M1 (Del KH1-2)**

ATGAACAAACTGTATATCGGAAACCTCAGCGAGAACGCCGCCCCCTCGGACCTAGAAAGTATCTTCAAGGACGCCAAGATCCCGGTGTCGGGACCCTTCCTGGTGAAGACTGGCTACGCGTTCGTGGACTGCCCGGACGAGAGCTGGGCCCTCAAGGCCATCGAGGCGCTTTCAGGTAAAATAGAACTGCACGGGAAACCCATAGAAGTTGAGCACTCGGTCCCAAAAAGGCAAAGGATTCGGAAACTTCAGATACGAAATATCCCGCCTCATTTACAGTGGGAGGTGCTGGATAGTTTACTAGTCCAGTATGGAGTGGTGGAGAGCTGTGAGCAAGTGAACACTGACTCGGAAACTGCAGTTGTAAATGTAACCTATTCCAGTAAGGACCAAGCTAGACAAGCACTAGACAAACTGAATGGATTTCAGTTAGAGAATTTCACCTTGAAAGTAGCCTATATCCCTGATGAAATGGCCGCCCAGCAAAACCCCTTGCAGCAGCCCCGAGGTCGCCGGGGGCTTGGGCAGAGGGGCTCCTCAAGGCAGGGGTCTCCAGGATCCGTATCCAAGCAGAAACCATGTCTGGAGATTATGCATAAGGAAGCTCAAGATATAAAATTCACAGAAATGAAGAAAATCAGGGAGTCTTATGAAAATGATATTGCTTCTATGAATCTTCAAGCACATTTAATTCCTGGATTAAATCTGAACGCCTTGGGTCTGTTCCCACCCACTTCAGGGATGCCACCTCCCACCTCAGGGCCCCCTTCAGCCATGACTCCTCCCTACCCGCAGTTTGAGCAATCAGAAACGGAGACTGTTCATCTGTTTATCCCAGCTCTATCAGTCGGTGCCATCATCGGCAAGCAGGGCCAGCACATCAAGCAGCTTTCTCGCTTTGCTGGAGCTTCAATTAAGATTGCTCCAGCGGAAGCACCAGATGCTAAAGTGAGGATGGTGATTATCACTGGACCACCAGAGGCTCAGTTCAAGGCTCAGGGAAGAATTTATGGAAAAATTAAAGAAGAAAACTTTGTTAGTCCTAAAGAAGAGGTGAAACTTGAAGCTCATATCAGAGTGCCATCCTTTGCTGCTGGCAGAGTTATTGGAAAAGGAGGCAAAACGGTGAATGAACTTCAGAATTTGTCAAGTGCAGAAGTTGTTGTCCCTCGTGACCAGACACCTGATGAGAATGACCAAGTGGTTGTCAAAATAACTGGTCACTTCTATGCTTGCCAGGTTGCCCAGAGAAAAATTCAGGAAATTCTGACTCAGGTAAAGCAGCACCAACAACAGAAGGCTCTGCAAAGTGGACCACCTCAGTCAAGACGGAAG

**IGF2BP3-M2 (Del KH3-4)**

ATGAACAAACTGTATATCGGAAACCTCAGCGAGAACGCCGCCCCCTCGGACCTAGAAAGTATCTTCAAGGACGCCAAGATCCCGGTGTCGGGACCCTTCCTGGTGAAGACTGGCTACGCGTTCGTGGACTGCCCGGACGAGAGCTGGGCCCTCAAGGCCATCGAGGCGCTTTCAGGTAAAATAGAACTGCACGGGAAACCCATAGAAGTTGAGCACTCGGTCCCAAAAAGGCAAAGGATTCGGAAACTTCAGATACGAAATATCCCGCCTCATTTACAGTGGGAGGTGCTGGATAGTTTACTAGTCCAGTATGGAGTGGTGGAGAGCTGTGAGCAAGTGAACACTGACTCGGAAACTGCAGTTGTAAATGTAACCTATTCCAGTAAGGACCAAGCTAGACAAGCACTAGACAAACTGAATGGATTTCAGTTAGAGAATTTCACCTTGAAAGTAGCCTATATCCCTGATGAAATGGCCGCCCAGCAAAACCCCTTGCAGCAGCCCCGAGGTCGCCGGGGGCTTGGGCAGAGGGGCTCCTCAAGGCAGGGGTCTCCAGGATCCGTATCCAAGCAGAAACCATGTGATTTGCCTCTGCGCCTGCTGGTTCCCACCCAATTTGTTGGAGCCATCATAGGAAAAGAAGGTGCCACCATTCGGAACATCACCAAACAGACCCAGTCTAAAATCGATGTCCACCGTAAAGAAAATGCGGGGGCTGCTGAGAAGTCGATTACTATCCTCTCTACTCCTGAAGGCACCTCTGCGGCTTGTAAGTCTATTCTGGAGATTATGCATAAGGAAGCTCAAGATATAAAATTCACAGAAGAGATCCCCTTGAAGATTTTAGCTCATAATAACTTTGTTGGACGTCTTATTGGTAAAGAAGGAAGAAATCTTAAAAAAATTGAGCAAGACACAGACACTAAAATCACGATATCTCCATTGCAGGAATTGACGCTGTATAATCCAGAACGCACTATTACAGTTAAAGGCAATGTTGAGACATGTGCCAAAGCTGAGGAGGAGATCATGAAGAAAATCAGGGAGTCTTATGAAAATGATATTGCTTCTATGAATCTTCAAGCACATTTAATTCCTGGATTAAATCTGAACGCCTTGGGTCTGTTCCCACCCACTTCAGGGATGCCACCTCCCACCTCAGGGCCCCCTTCAGCCATGACTCCTCCCTACCCGCAGTTTGAGCAATCAGAATATGGAAAAATTAAAGAAGAAAACTTTGTTAGTCCTAAAGAAGAGGTGCAGGAAATTCTGACTCAGGTAAAGCAGCACCAACAACAGAAGGCTCTGCAAAGTGGACCACCTCAGTCAAGACGGAAG

**IGF2BP3-M3 (Del RRM1-2)**

ATGAAAAGGCAAAGGATTGAAATGGCCGCCCAGCAAAACCCCTTGCAGCAGCCCCGAGGTCGCCGGGGGCTTGGGCAGAGGGGCTCCTCAAGGCAGGGGTCTCCAGGATCCGTATCCAAGCAGAAACCATGTGATTTGCCTCTGCGCCTGCTGGTTCCCACCCAATTTGTTGGAGCCATCATAGGAAAAGAAGGTGCCACCATTCGGAACATCACCAAACAGACCCAGTCTAAAATCGATGTCCACCGTAAAGAAAATGCGGGGGCTGCTGAGAAGTCGATTACTATCCTCTCTACTCCTGAAGGCACCTCTGCGGCTTGTAAGTCTATTCTGGAGATTATGCATAAGGAAGCTCAAGATATAAAATTCACAGAAGAGATCCCCTTGAAGATTTTAGCTCATAATAACTTTGTTGGACGTCTTATTGGTAAAGAAGGAAGAAATCTTAAAAAAATTGAGCAAGACACAGACACTAAAATCACGATATCTCCATTGCAGGAATTGACGCTGTATAATCCAGAACGCACTATTACAGTTAAAGGCAATGTTGAGACATGTGCCAAAGCTGAGGAGGAGATCATGAAGAAAATCAGGGAGTCTTATGAAAATGATATTGCTTCTATGAATCTTCAAGCACATTTAATTCCTGGATTAAATCTGAACGCCTTGGGTCTGTTCCCACCCACTTCAGGGATGCCACCTCCCACCTCAGGGCCCCCTTCAGCCATGACTCCTCCCTACCCGCAGTTTGAGCAATCAGAAACGGAGACTGTTCATCTGTTTATCCCAGCTCTATCAGTCGGTGCCATCATCGGCAAGCAGGGCCAGCACATCAAGCAGCTTTCTCGCTTTGCTGGAGCTTCAATTAAGATTGCTCCAGCGGAAGCACCAGATGCTAAAGTGAGGATGGTGATTATCACTGGACCACCAGAGGCTCAGTTCAAGGCTCAGGGAAGAATTTATGGAAAAATTAAAGAAGAAAACTTTGTTAGTCCTAAAGAAGAGGTGAAACTTGAAGCTCATATCAGAGTGCCATCCTTTGCTGCTGGCAGAGTTATTGGAAAAGGAGGCAAAACGGTGAATGAACTTCAGAATTTGTCAAGTGCAGAAGTTGTTGTCCCTCGTGACCAGACACCTGATGAGAATGACCAAGTGGTTGTCAAAATAACTGGTCACTTCTATGCTTGCCAGGTTGCCCAGAGAAAAATTCAGGAAATTCTGACTCAGGTAAAGCAGCACCAACAACAGAAGGCTCTGCAAAGTGGACCACCTCAGTCAAGACGGAAG

**IGF2BP3-M4 (Del KH1-4)**

ATGAACAAACTGTATATCGGAAACCTCAGCGAGAACGCCGCCCCCTCGGACCTAGAAAGTATCTTCAAGGACGCCAAGATCCCGGTGTCGGGACCCTTCCTGGTGAAGACTGGCTACGCGTTCGTGGACTGCCCGGACGAGAGCTGGGCCCTCAAGGCCATCGAGGCGCTTTCAGGTAAAATAGAACTGCACGGGAAACCCATAGAAGTTGAGCACTCGGTCCCAAAAAGGCAAAGGATTCGGAAACTTCAGATACGAAATATCCCGCCTCATTTACAGTGGGAGGTGCTGGATAGTTTACTAGTCCAGTATGGAGTGGTGGAGAGCTGTGAGCAAGTGAACACTGACTCGGAAACTGCAGTTGTAAATGTAACCTATTCCAGTAAGGACCAAGCTAGACAAGCACTAGACAAACTGAATGGATTTCAGTTAGAGAATTTCACCTTGAAAGTAGCCTATATCCCTGATGAAATGGCCGCCCAGCAAAACCCCTTGCAGCAGCCCCGAGGTCGCCGGGGGCTTGGGCAGAGGGGCTCCTCAAGGCAGGGGTCTCCAGGATCCGTATCCAAGCAGAAACCATGTCTGGAGATTATGCATAAGGAAGCTCAAGATATAAAATTCACAGAAATGAAGAAAATCAGGGAGTCTTATGAAAATGATATTGCTTCTATGAATCTTCAAGCACATTTAATTCCTGGATTAAATCTGAACGCCTTGGGTCTGTTCCCACCCACTTCAGGGATGCCACCTCCCACCTCAGGGCCCCCTTCAGCCATGACTCCTCCCTACCCGCAGTTTGAGCAATCAGAATATGGAAAAATTAAAGAAGAAAACTTTGTTAGTCCTAAAGAAGAGGTGCAGGAAATTCTGACTCAGGTAAAGCAGCACCAACAACAGAAGGCTCTGCAAAGTGGACCACCTCAGTCAAGACGGAAG

**The wild-type (WT) and mutant (M1-M3) reporter plasmid sequences of DDX21 were exhibited as follow.**

**DDX21-WT**

TCTCTGTGGCCACAGAGCAACCAGAACTGGAAGGACCACGGGAAGGATATGGAGGCTTCAGGGGACAGCGGGAAGGCAGTCGAGGCTTCAGGGGACAGCGGGACGGAAACAGAAGATTCAGAGGACAGCGGGAAGGCAGTAGAGGCCCGAGAGG**A**CAGCGATCAGGAGGTGGCAACAAAAGTAACAGATCCCAAA**A**CAAAGGCCAGAAGCGGAGTTTCAGTAAAGCATTTGGTCAATAATTAGAAATAGAAGATTTATATAGCAAAAAGAGAATGATGTTTGGCAATATAGA**A**CTGA**A**CATTATTTTTCATGCAAAGTTAAAAGCACATTGTGCCTCCTTTTGACCACTTGCCAAGTCCCTGTCTCTTTCAG**A**CACAG**A**CAAGCTTCATTTAAATTATTTCATCTGATCATTATCATTTATAACTTTATTGTTA

**DDX21-M1**

TCTCTGTGGCCACAGAGCAACCAGAACTGGAAGGACCACGGGAAGGATATGGAGGCTTCAGGGGACAGCGGGAAGGCAGTCGAGGCTTCAGGGGACAGCGGGACGGAAACAGAAGATTCAGAGGACAGCGGGAAGGCAGTAGAGGCCCGAGAGGACAGCGATCAGGAGGTGGCAACAAAAGTAACAGATCCCAAAACAAAGGCCAGAAGCGGAGTTTCAGTAAAGCATTTGGTCAATAATTAGAAATAGAAGATTTATATAGCAAAAAGAGAATGATGTTTGGCAATATAGA**C**CTGA**C**CATTATTTTTCATGCAAAGTTAAAAGCACATTGTGCCTCCTTTTGACCACTTGCCAAGTCCCTGTCTCTTTCAGACACAGACAAGCTTCATTTAAATTATTTCATCTGATCATTATCATTTATAACTTTATTGTTA

**DDX21-M2**

TCTCTGTGGCCACAGAGCAACCAGAACTGGAAGGACCACGGGAAGGATATGGAGGCTTCAGGGGACAGCGGGAAGGCAGTCGAGGCTTCAGGGGACAGCGGGACGGAAACAGAAGATTCAGAGGACAGCGGGAAGGCAGTAGAGGCCCGAGAGGACAGCGATCAGGAGGTGGCAACAAAAGTAACAGATCCCAAAACAAAGGCCAGAAGCGGAGTTTCAGTAAAGCATTTGGTCAATAATTAGAAATAGAAGATTTATATAGCAAAAAGAGAATGATGTTTGGCAATATAGA**C**CTGA**C**CATTATTTTTCATGCAAAGTTAAAAGCACATTGTGCCTCCTTTTGACCACTTGCCAAGTCCCTGTCTCTTTCAG**C**CACAG**C**CAAGCTTCATTTAAATTATTTCATCTGATCATTATCATTTATAACTTTATTGTTA

**DDX21-M3**

TCTCTGTGGCCACAGAGCAACCAGAACTGGAAGGACCACGGGAAGGATATGGAGGCTTCAGGGGACAGCGGGAAGGCAGTCGAGGCTTCAGGGGACAGCGGGACGGAAACAGAAGATTCAGAGGACAGCGGGAAGGCAGTAGAGGCCCGAGAGG**C**CAGCGATCAGGAGGTGGCAACAAAAGTAACAGATCCCAAA**C**CAAAGGCCAGAAGCGGAGTTTCAGTAAAGCATTTGGTCAATAATTAGAAATAGAAGATTTATATAGCAAAAAGAGAATGATGTTTGGCAATATAGA**C**CTGA**C**CATTATTTTTCATGCAAAGTTAAAAGCACATTGTGCCTCCTTTTGACCACTTGCCAAGTCCCTGTCTCTTTCAG**C**CACAG**C**CAAGCTTCATTTAAATTATTTCATCTGATCATTATCATTTATAACTTTATTGTTA

**Supplementary Figures and corresponding Figure Legends**

**
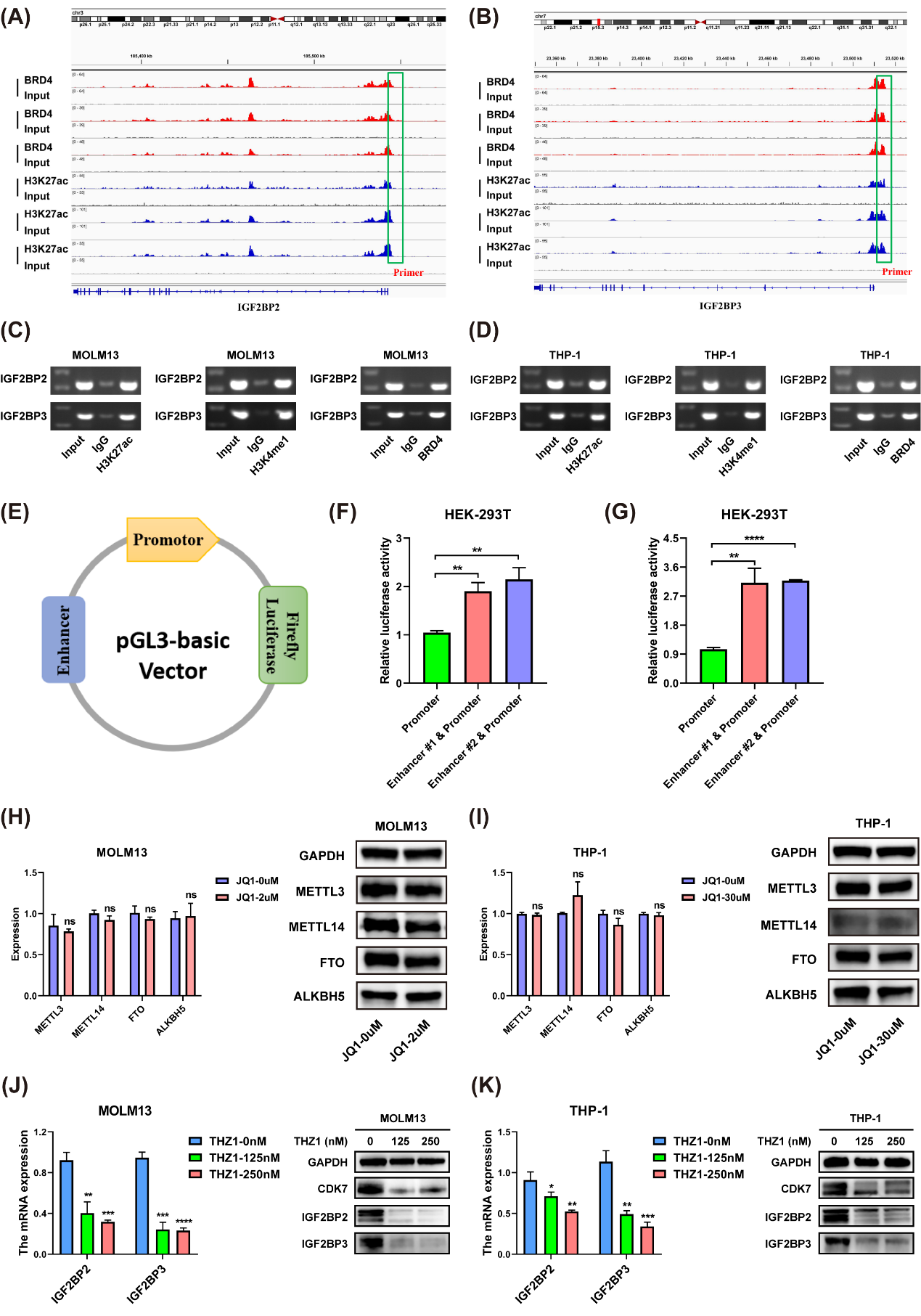
**

**FIGURE S1** IGF2BP2 and IGF2BP3 were SE-driven genes. **(A and B)** The qPCR primers for ChIP experiments using H3K27ac, H3K4me1 and BRD4 antibodies were designed based on the location of SE regions on IGF2BP2 and IGF2BP3 (the specific sequence regions were shown in the green box). **(C and D)** DNA gel electrephoresis assays were carried out using the products from ChIP-qPCR assays with antibodies against H3K27ac, H3K4me1 and BRD4, respectively. **(E)** Schematic diagram of Enhancer & Promoter plasmids. Diverse enhancers and promoter were inserted into pGL3-basic vector assembled with firefly luciferase. These Enhancer & Promoter plasmids and renilla plasmids were cotransfected into HEK-293T cells, followed by the luciferase reporter assays. **(F and G)** Luciferase activities of each group (Promoter, Enhancer#1 & Promoter and Enhancer#2 & Promoter) of IGF2BP2 **(F)** and IGF2BP3 **(G)** were determined. (**H and I**) Expression of m^6^A enzymes METTL3, METTL14, FTO and ALKBH5 were detected when AML cells were treated with JQ1. **(J and K)** After THZ1 treatment, IGF2BP2 and IGF2BP3 expression were detected.


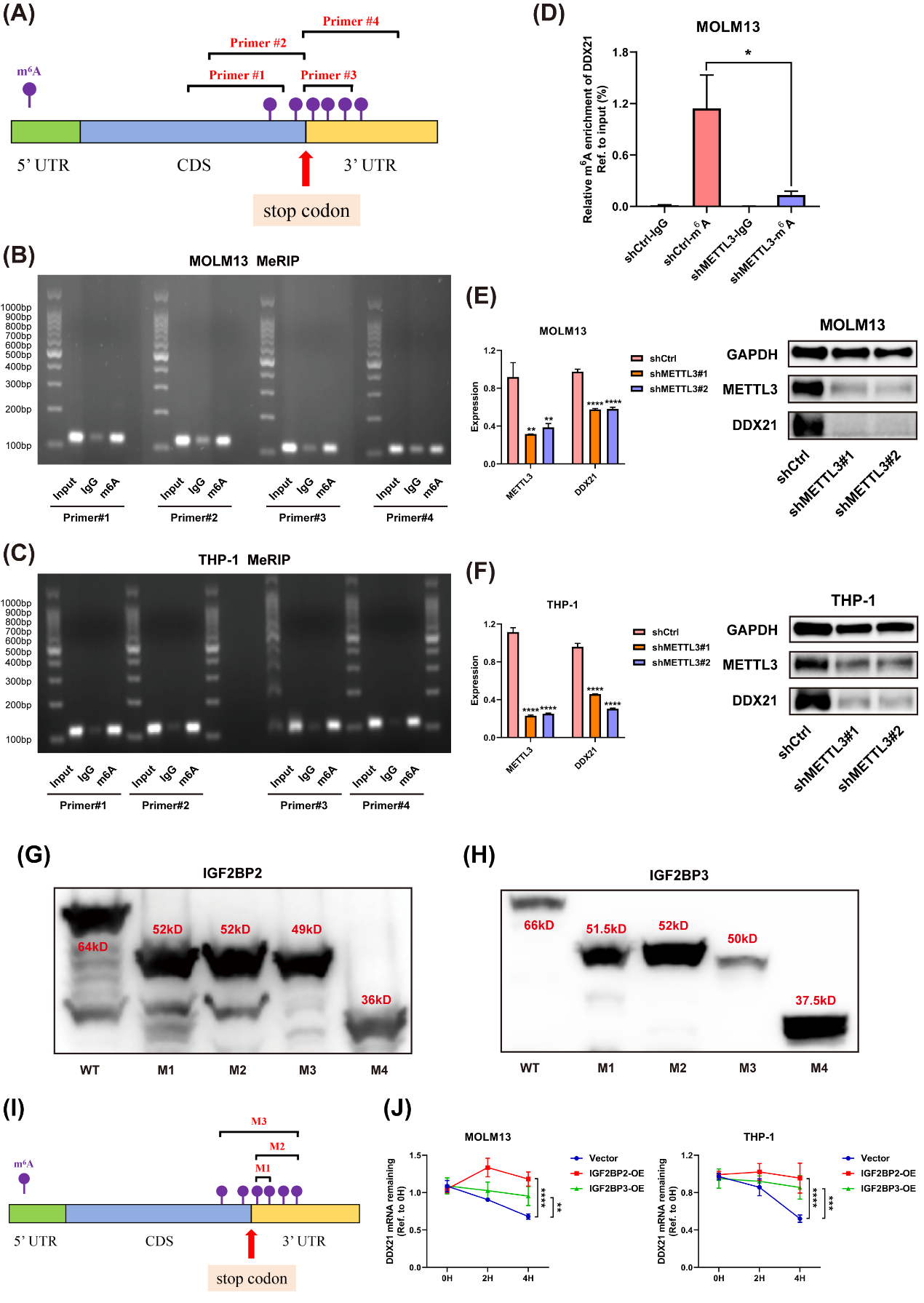


**FIGURE S2** DDX21 was modified by m^6^A and recognized by IGF2BP2/3. **(A)** With the help of RMBase v2.0 (http://rna.sysu.edu.cn/rmbase/) and SRAMP (http://www.cuilab.cn/sramp), the possible m^6^A sites on DDX21 were predicted to be mainly located in the CDS near the stop codon and 3’ UTR. Then we designed qPCR primers to ensure that target sequence contained as many of these sites as possible. The primer#1 and 2 focused on the sites in CDS near the stop codon, while the primer#3 and 4 focused on the sites in 3’ UTR. **(B and C)** The results of DNA gel electrephoresis assays using the products from MeRIP-qPCR assays were displayed. The length of products was approximately 100bp, and the signal of m^6^A group was more intensive than IgG group. **(D)** The results of MeRIP-qPCR assay when METTL3 was knockdown in MOLM13 cells. **(E and F)** Expression of DDX21 was detected after METTL3 was silenced. **(G and H)** After 293T cells were transfected with WT and truncation plasmids (M1-4), western blotting assays validated the relative molecular weight (MW) of corresponding proteins. For WT and M1-4 groups of IGF2BP2, the MW was 64kD, 52kD, 52kD, 49kD and 36kD, respectively. For IGF2BP3, the MW was 66kD, 51.5kD, 52kD, 50kD and 37.5kD, respectively. **(I)** Sketch-map of the design for m^6^A-related luciferase reporter plasmids. Six potential m^6^A sites were shown in CDS and 3’UTR around stop codon. For M1, M2 and M3 plasmids, two, four and six m^6^A sites were mutated, respectively. **(J)** RNA decay assays were performed when IGF2BP2 or IGF2BP3 was overexpressed.

**
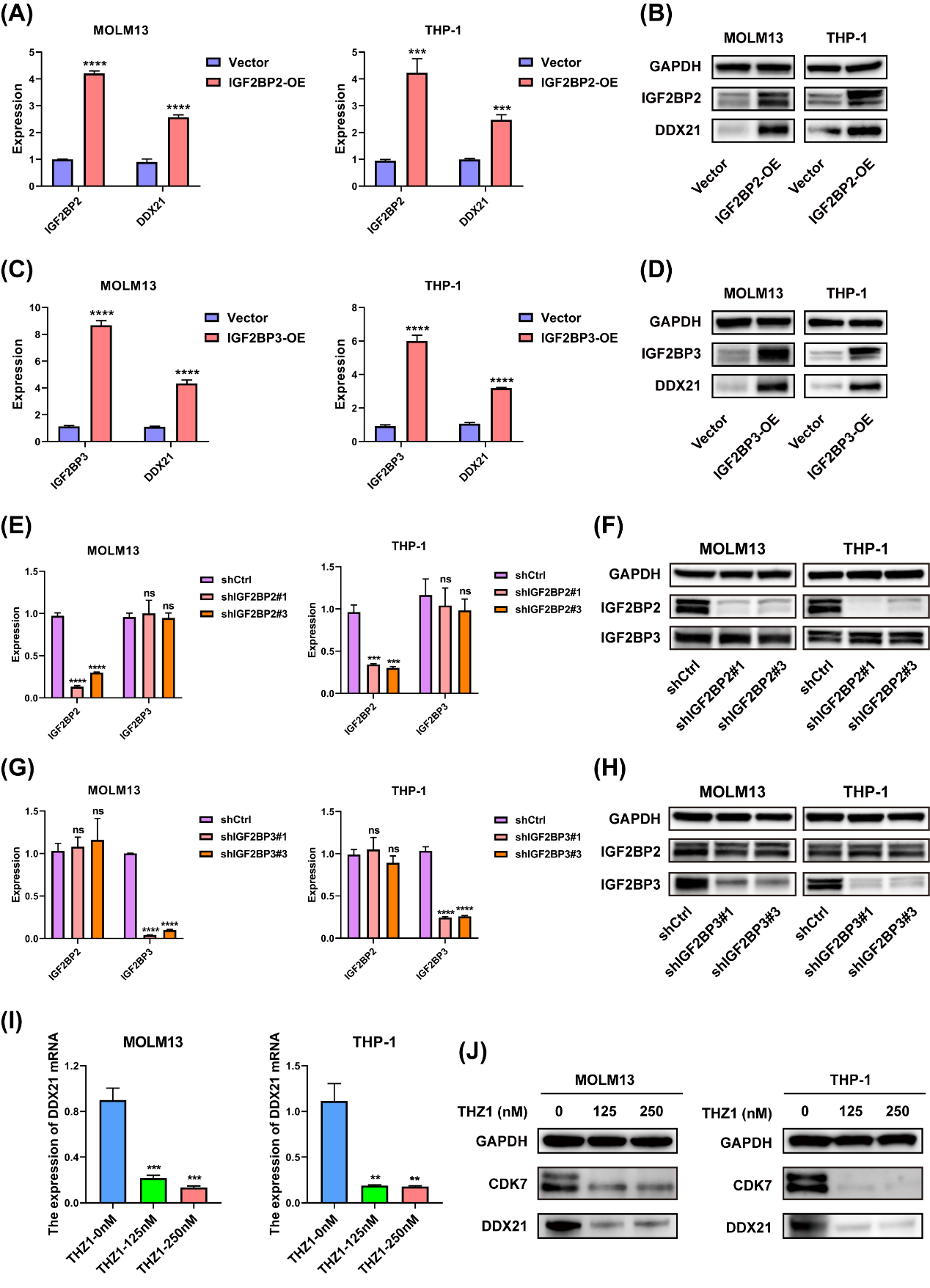
**

**FIGURE S3** DDX21 was regulated by SE-triggered IGF2BP2/3. **(A-D)** The changes of DDX21 expression were determined when IGF2BP2 **(A and B)** or IGF2BP3 **(C and D)** was overexpressed. **(E and F)** The expression of IGF2BP3 was detected after the knockdown of IGF2BP2. **(G and H)** The expression of IGF2BP2 was detected after the knockdown of IGF2BP3. **(I and J)** The impacts of THZ1 on DDX21 expression were examined via RT-qPCR (**I**) and western blotting (**J**) assays.


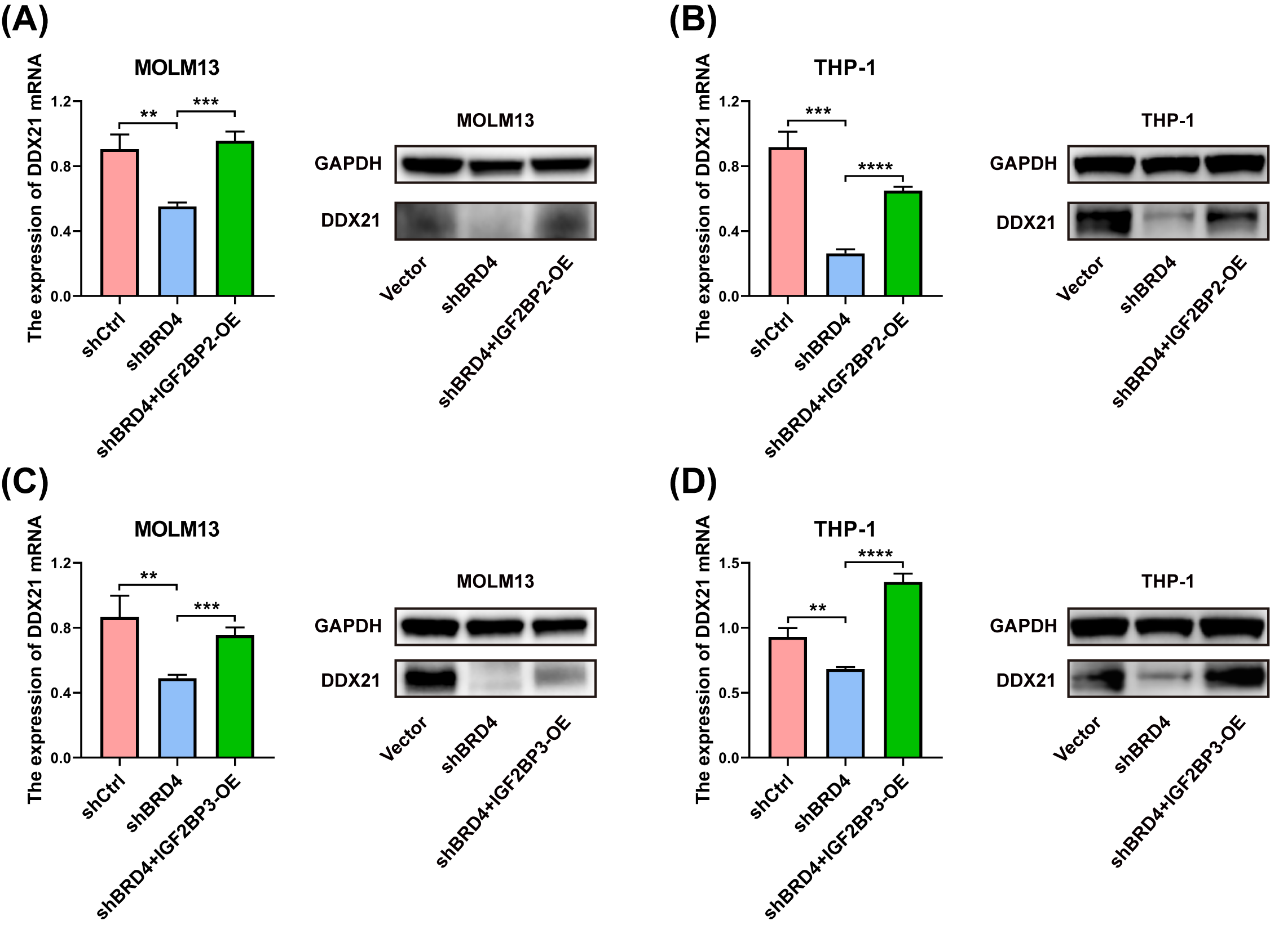


**FIGURE S4** IGF2BP2 or IGF2BP3 overexpression reversed the inhibitory effects of BRD4 silencing on DDX21. **(A-D)** The rescue models were established containing three groups. MOLM13 and THP-1 cells were transfected with empty vector as the control group. One experimental group was transfected with BRD4-silencing lentiviruses, another was successively transfected with BRD4-knockdown and IGF2BP2/IGF2BP3-overexpression lentiviruses. The expression of DDX21 was measured to evaluate the rescue effects of IGF2BP2 (**A and B**) or IGF2BP3 (**C and D**) overexpression.


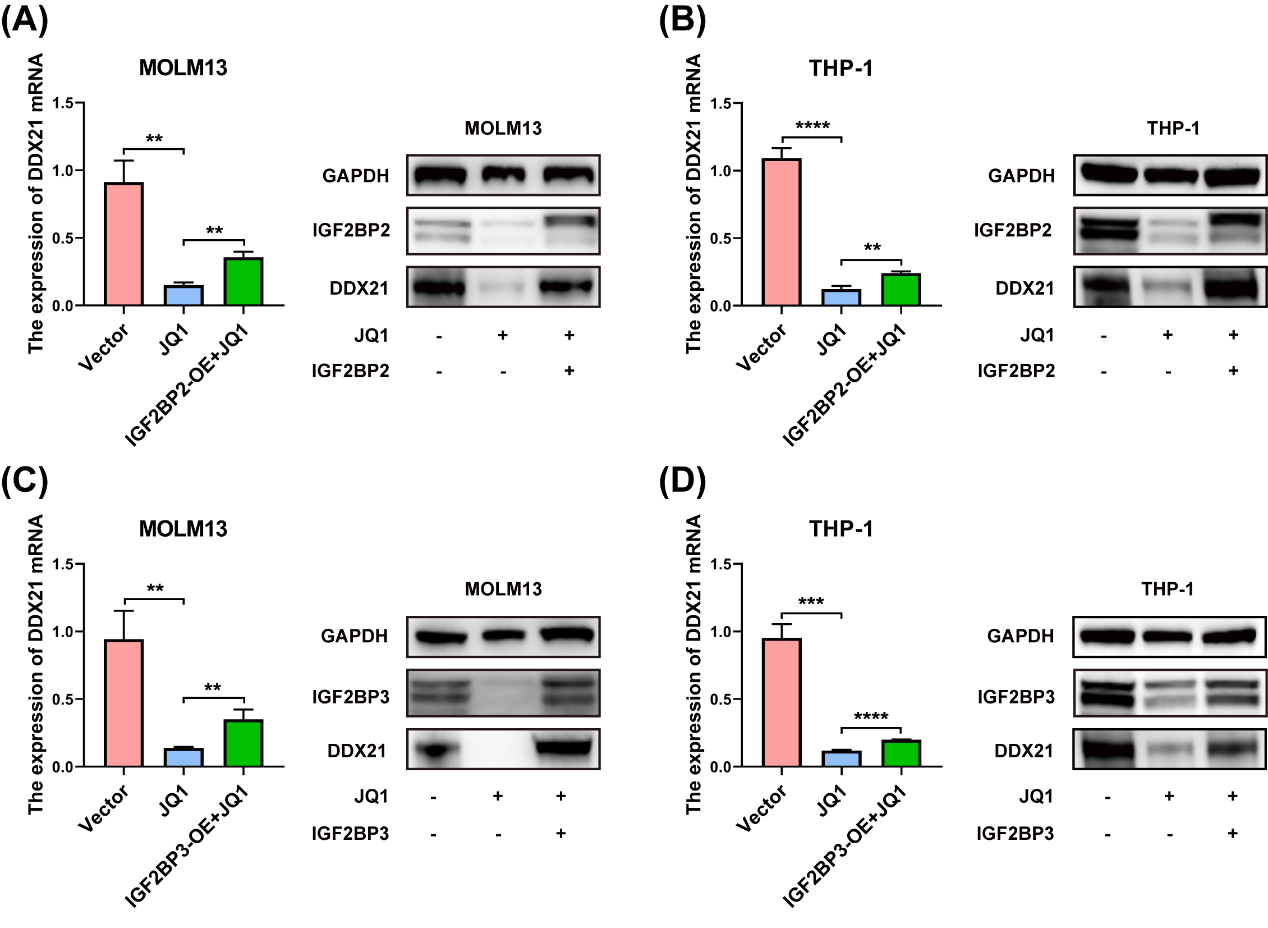


**FIGURE S5** IGF2BP2 or IGF2BP3 overexpression reversed the inhibitory effects of JQ1 treatment on DDX21. **(A-D)** Rescue models with the treatment of JQ1 were designed, including control group, JQ1 group, and JQ1 combined with IGF2BP2/IGF2BP3-overexpression group. The changes in the DDX21 expression were examined to evaluate the rescue effects of IGF2BP2 (**A and B**) or IGF2BP3 (**C and D**) overexpression.

**
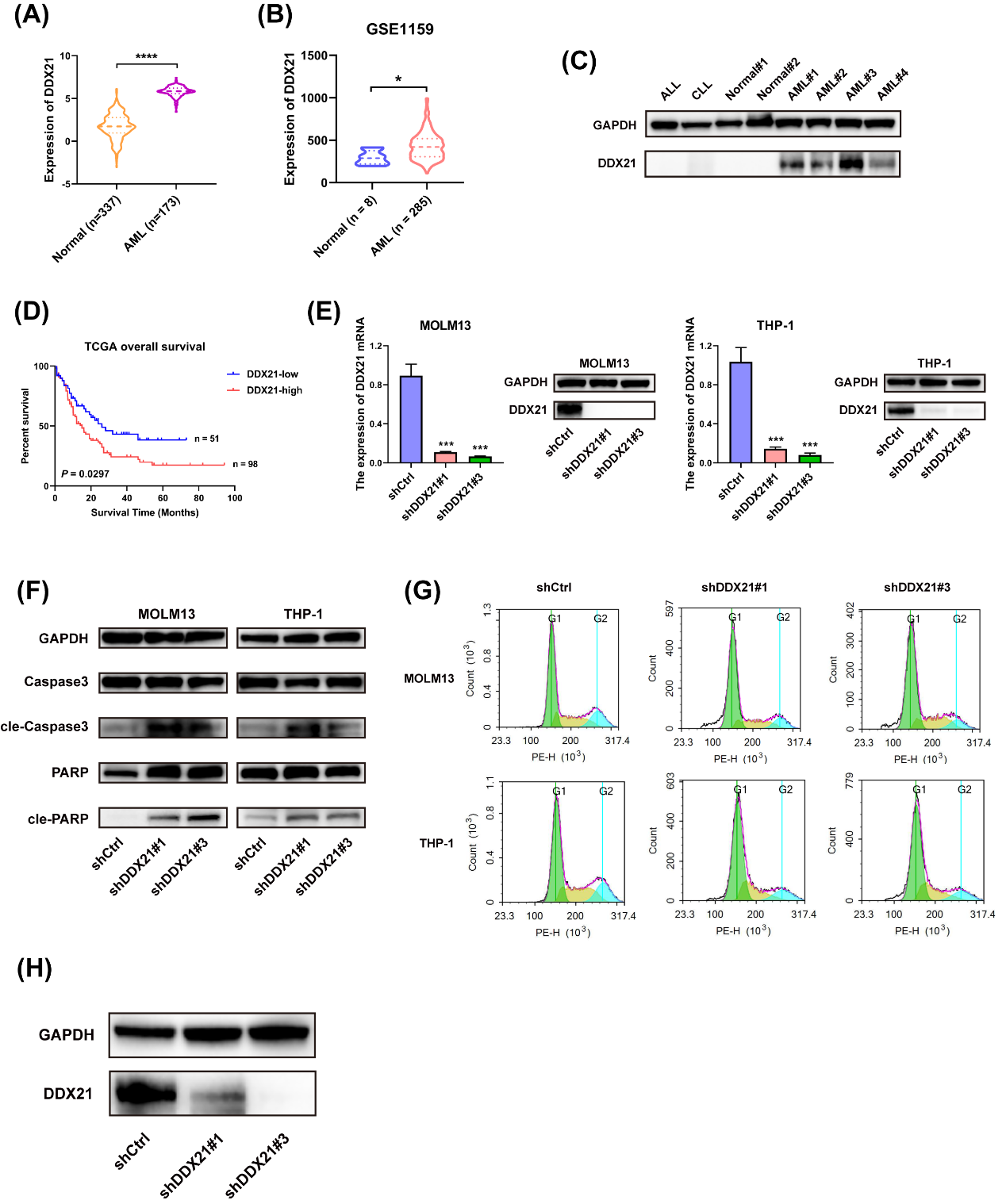
**

**FIGURE S6** DDX21 was highly expressed in AML, regulating cell apoptosis and cell cycle. **(A)** DDX21 expression in AML patients (n=173, from TCGA database) was compared with that in normal people (n=337, from GTEx database). **(B)** The expression of DDX21 was compared between normal (n=8) and AML (n=285) patients from the GEO cohort (GSE1159). **(C)** The protein levels of DDX21 in ALL (n=1), CLL (n=1), normal (n=2) and AML (n=4) primary cells were measured, respectively. **(D)** The overall survival of AML patients from TCGA database was assessed via Kaplan-Meier analysis. **(E)** Knockdown efficiency of DDX21 was determined. **(F)** Expression of apoptosis-related proteins in AML cells were detected via western blotting assays when DDX21 was knocked down. **(G)** Cell cycle assays were performed in AML cells with DDX21 knockdown or not. **(H)** Knockdown efficiency of DDX21 from *in vivo* experiment was determined.


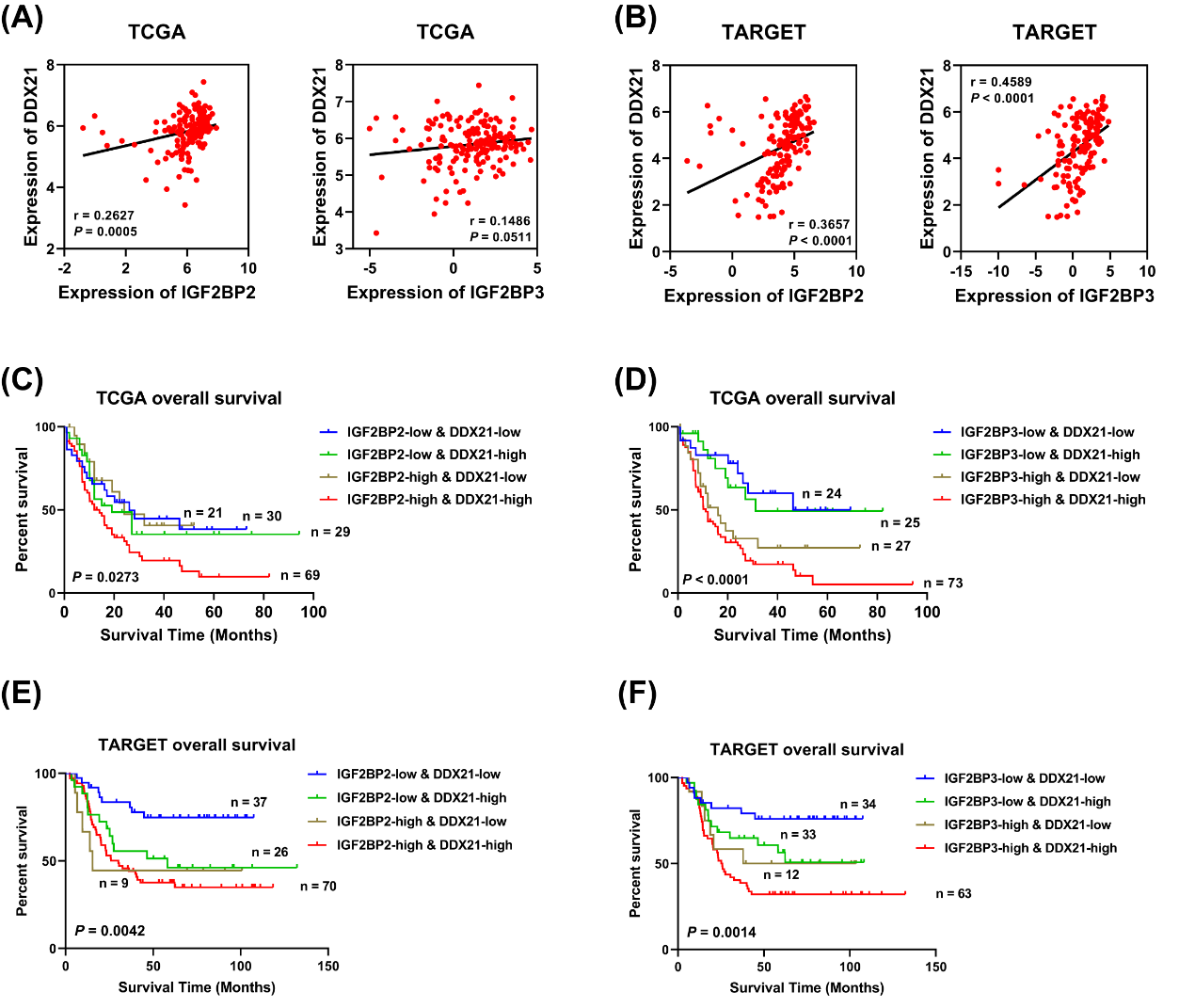


**FIGURE S7** Co-expression and survival analysis of DDX21 were performed. **(A and B)** The expression between IGF2BP2 or IGF2BP3 and DDX21 was analyzed based on TCGA (**A**) and TARGET (**B**) databases. **(C-F)** According to the expression of DDX21 and IGF2BP2 or IGF2BP3 from TCGA (**C and D**) and TARGET (**E and F**) databases, AML patients were divided into four groups, respectively. Following Kaplan-Meier survival analysis was conducted.


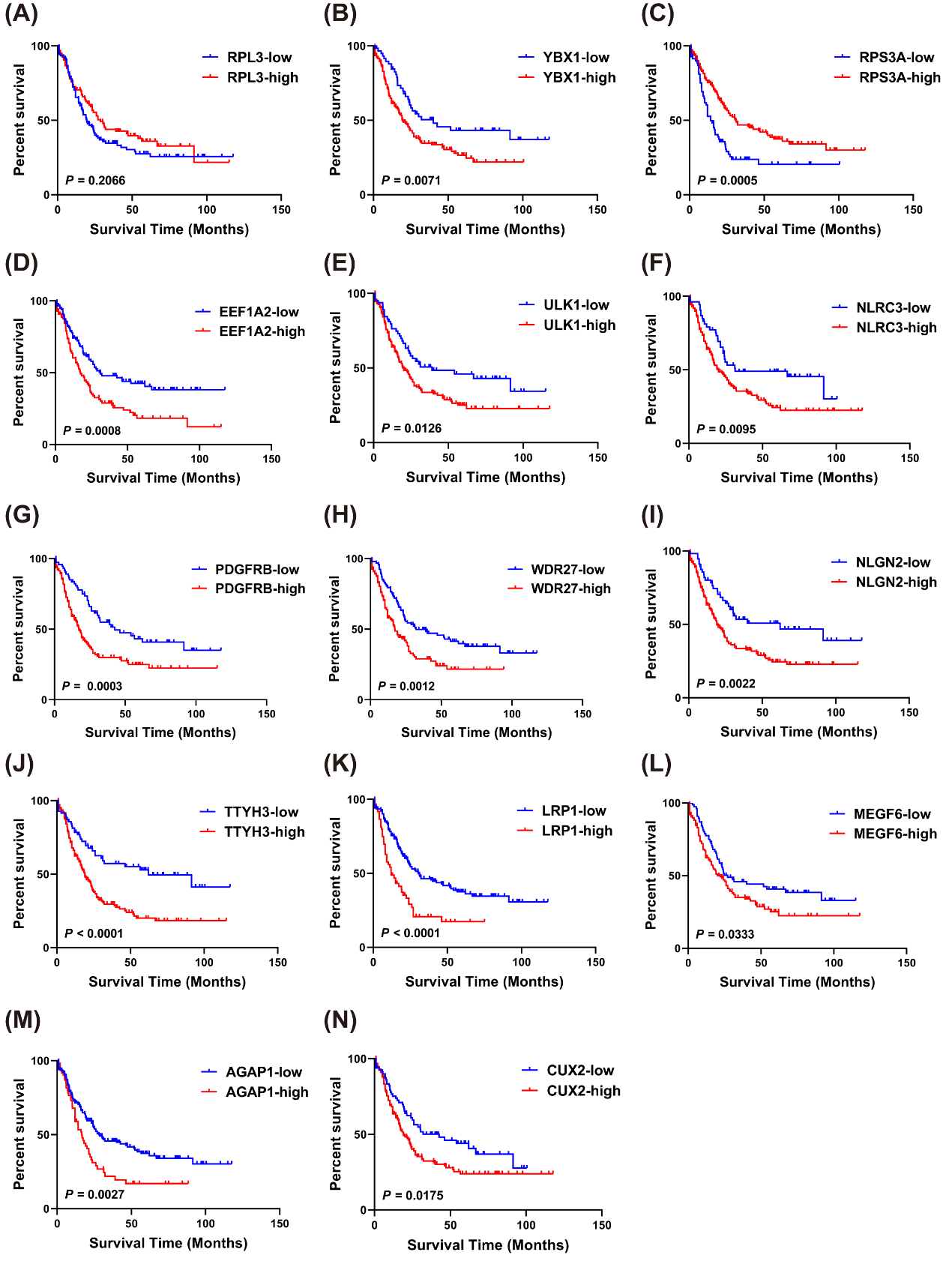


**FIGURE S8** Survival analysis of AML patients based on the possible DDX21-binding proteins and the potential targets of DDX21. **(A-C)** According to TCGA database, overall survival analysis of three possible binding proteins of DDX21 including RPL3 (**A**), YBX1 (**B**) and RPS3A (**C**) were performed. **(D-N)** The survival analysis of 21 genes selected from the Figure 4E were evaluated based on TCGA database. Finally, elevated expression of 11 genes, including EEF1A2 (**D**), ULK1 (**E**), NLRC3 (**F**), PDGFRB (**G**), WDR27 (**H**), NLGN2 (**I**), TTYH3 (**J**), LRP1 (**K**), MEGF6 (**L**), AGAP1 (**M**) and CUX2 (**N**), suggested the poor survival of AML patients.


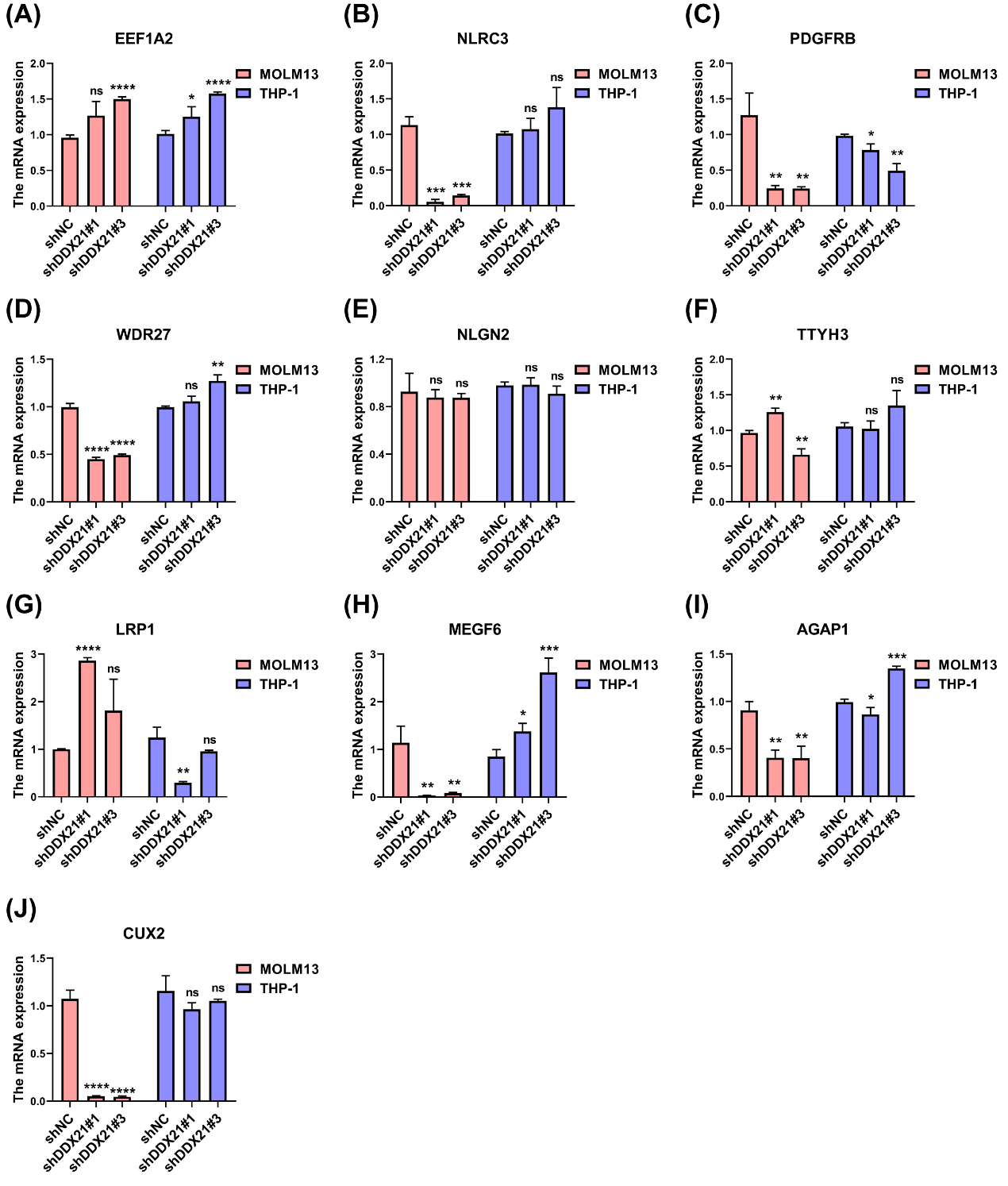


**FIGURE S9** The impacts of DDX21 silencing on the expression of potential downstream transcripts. **(A-J)** After DDX21 was knocked down in MOLM13 and THP-1 cells, the expression of 10 candidate genes were detected via RT-qPCR assays. These transcripts included EEF1A2 (**A**), NLRC3 (**B**), PDGFRB (**C**), WDR27 (**D**), NLGN2 (**E**), TTYH3 (**F**), LRP1 (**G**), MEGF6 (**H**), AGAP1 (**I**) and CUX2 (**J**).


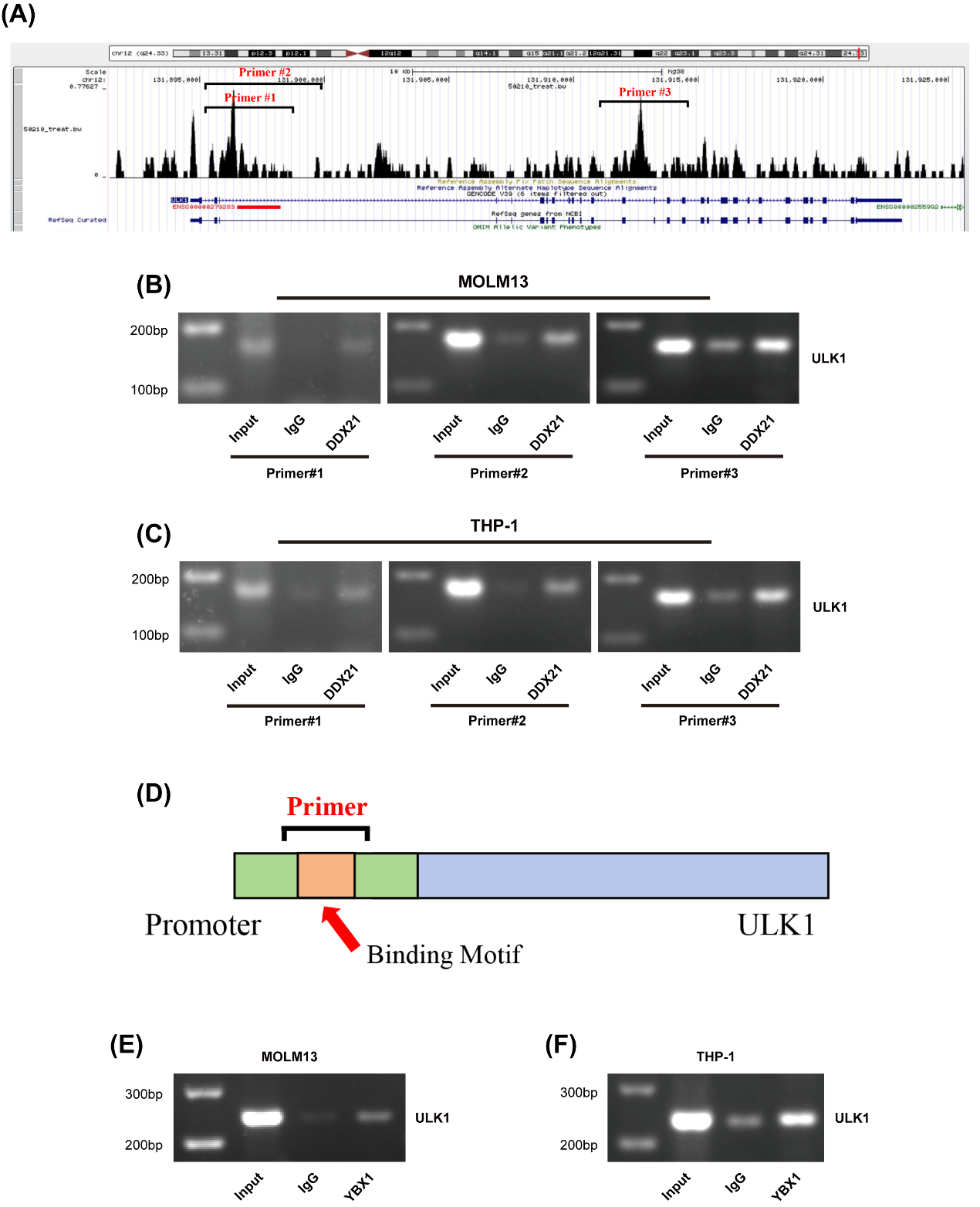


**FIGURE S10** The binding of DDX21 and YBX1 to ULK1. **(A)** With the help of public ChIP-seq data of DDX21 (GSE56802), we obtained two possible binding sites of DDX21 on ULK1 (the peaks), and designed the corresponding qPCR primers (the primer#1 and 2 focused on the sites in the first peak, while the primer#3 focused on the sites in the second peak). **(B and C)** DNA gel electrephoresis assays were conducted using the products from ChIP-qPCR assays using DDX21 antibody. **(D)** The suitable primer for ChIP-qPCR assays using YBX1 antibody was designed with the help of JASPAR (http://jaspar.genereg.net/) which could predict the possible binding sites of YBX1 on ULK1 promoter. **(E and F)** DNA gel electrephoresis assays were performed utilizing the products of ChIP-qPCR assays using YBX1 antibody.

**
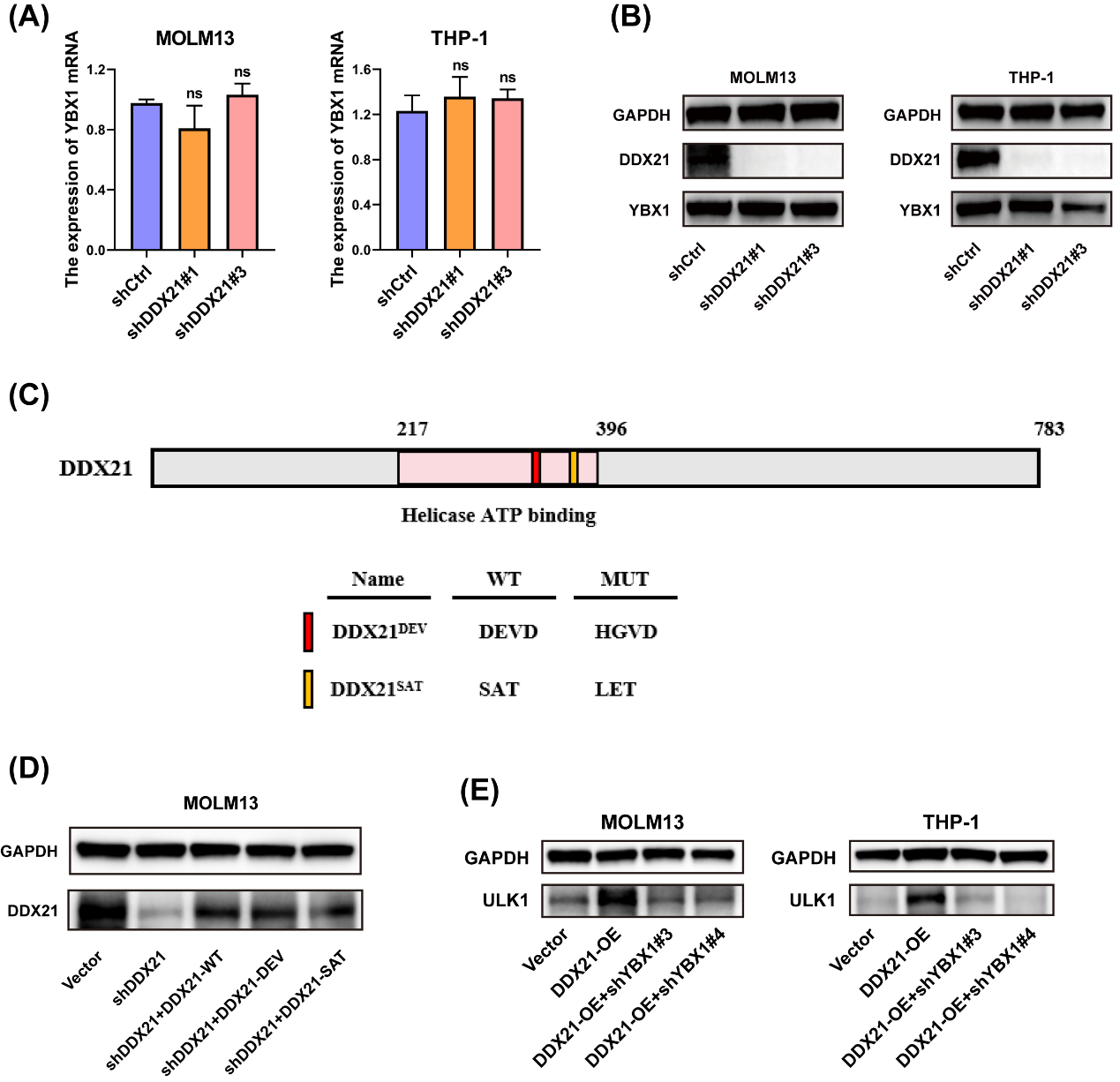
**

**FIGURE S11** The regulation of DDX21 for YBX1 and ULK1. **(A and B)** Expression impacts of DDX21 on YBX1 were determined. **(C)** Schematic illustration of enzymatic activity mutation of DDX21. Helicase ATP binding sites in DDX21 were mutated, including the transformation from DEVD to HGVD (DDX21^DEV^) and from SAT to LET (DDX21^SAT^) , respectively. (**D**) Protein levels of DDX21 were measured when mutated DDX21 plasmids were transfected into DDX21-silenced cells. **(E)** Rescue models were designed, including control group, DDX21-overexpression group, and DDX21-overexpression combined with YBX1-knockdown group. The alterations of ULK1 expression were examined.

**
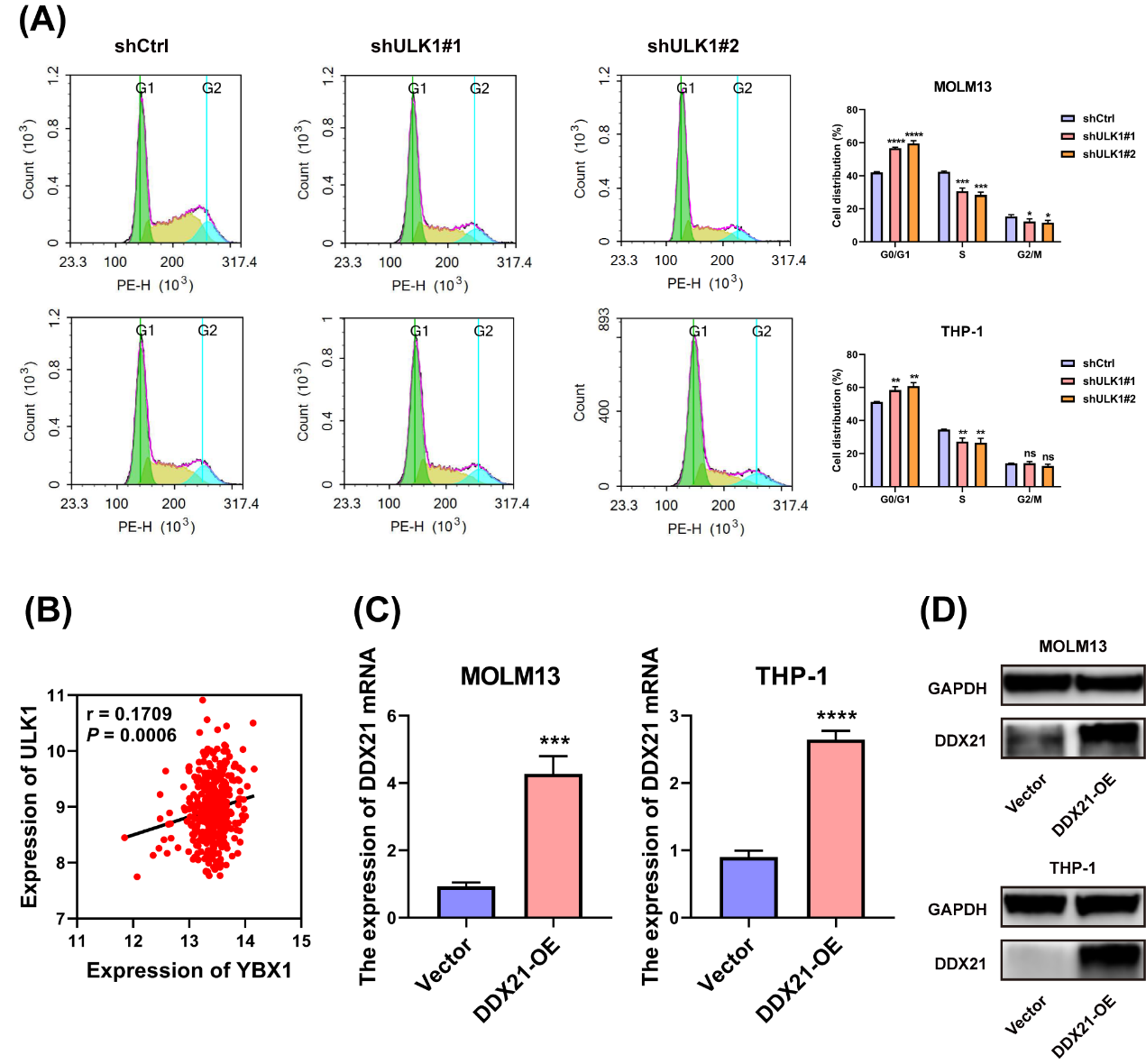
**

**FIGURE S12** The functions of ULK1 and overexpression efficiency of DDX21. **(A)** Cell cycle assays were performed in AML cells when ULK1 was knocked down. **(B)** The relationship between YBX1 and ULK1 expression was analyzed based on GEO cohort (GSE37642). **(C and D)** The efficiency of DDX21 overexpression was detected at RNA (**C**) and protein (**D**) levels.

**Supplementary Tables**

**TABLE S1 Targeted sequences of shRNAs in this work.**

| Gene symbol | Targeted sequence |
| --- | --- |
| shBRD4#1 | GGAAGTGGAAGAGAATAAA |
| shBRD4#2 | GATTACTATAAGATCATTA |
| shIGF2BP2#1 | CGAAGAGATTCCTCTGAAA |
| shIGF2BP2#3 | GGCAGATGAGACCAAACTA |
| shIGF2BP3#1 | GGTGCTGGATAGTTTACTA |
| shIGF2BP3#3 | GGTAAAGCAGCACCAACAA |
| shDDX21#1 | GGTTGATTTGGTTATACAA |
| shDDX21#3 | GAAAGTGGTTTCTTCTAAA |
| shYBX1#3 | TTGACACCAAGGAAGATGT |
| shYBX1#4 | TGTGAGAGTGGGGAAAAGA |
| shULK1#1 | CAAGCACTTTATGCATATA |
| shULK1#2 | CGCGGTACCTCCAGAGCAA |
| shMETTL3#1 | GCCAAGGAACAATCCATTG |
| shMETTL3#2 | GCTGCACTTCAGACGAATT |

**TABLE S2 Sequences of primers in this study.**

| Gene symbol | Forward primer | Reverse primer |
| --- | --- | --- |
| GAPDH | GGAGCGAGATCCCTCCAAAAT | GGCTGTTGTCATACTTCTCATGG |
| DDX21 | GAGGAGCCATCTCAAAATGACA | GGGTTACAGTCCGGTTCAGG |
| BRD4 | ACCTCCAACCCTAACAAGCC | TTTCCATAGTGTCTTGAGCACC |
| IGF2BP2 | AGCTAAGCGGGCATCAGTTTG | CCGCAGCGGGAAATCAATCT |
| IGF2BP3 | TATATCGGAAACCTCAGCGAGA | GGACCGAGTGCTCAACTTCT |
| METTL3 | CAAGCTGCACTTCAGACGAA | GCTTGGCGTGTGGTCTTT |
| METTL14 | AGAAACTTGCAGGGCTTCCT | TCTTCTTCATATGGCAAATTTTCTT |
| FTO | ACTTGGCTCCCTTATCTGACC | TGTGCAGTGTGAGAAAGGCTT |
| ALKBH5 | CGGCGAAGGCTACACTTACG | CCACCAGCTTTTGGATCACCA |
| ULK1 | GGCAAGTTCGAGTTCTCCCG | CGACCTCCAAATCGTGCTTCT |
| IGF2BP2-ChIP | TTGGCAGCTGCTCTCTATTCT | GTCGGTCACGTTGTTTCCTC |
| IGF2BP3-ChIP | TTCCCTGCCCATTTCCAACA | ACGAAAACGTGGTCTCAGCA |
| ULK1-ChIP (YBX1) | CACTTACCTCGCAGCGTGT | TTTTACCTGCACCCAGCTCAT |
| ULK1-ChIP-1 (DDX21) | GCTCTGCGTCAGACGGG | GAAAGCCAGCTGGTCCCAC |
| ULK1-ChIP-2 (DDX21) | GTGTCTGCCAGGGACACAAT | CTACCCCTGGTGTCCGTCTT |
| ULK1-ChIP-3 (DDX21) | CCACGTGTTTGTTGAATGAAGGT | GAGTCATTGTGTCCCTGGCA |
| DDX21-MeRIP-1 | GCCACAGAGCAACCAGAACT | ATCTTCTGTTTCCGTCCCGC |
| DDX21-MeRIP-2 | GAACTGGAAGGACCACGGG | TCCGCTTCTGGCCTTTGTTT |
| DDX21-MeRIP-3 | CAAAAAGAGAATGATGTTTGGCAAT | GTGTCTGAAAGAGACAGGGACT |
| DDX21-MeRIP-4 | TGTTTGGCAATATAGAACTGAACAT | TGTGTCTGAAAGAGACAGGGA |
| TTYH3 | AGAACGCTAATTTCCAGAACCC | GTGGCGAGGTATTTGGCTCTC |
| EEF1A2 | GTCAAGGAAGTCAGCGCCTAC | TGAACCACGGCATGTTGGG |
| NLRC3 | GTGCCGACCGACTCATCTG | GTCCTGCACTCATCCAAGC |
| WDR27 | CACAAGAAGGGTTAAGTCTGGG | GGTGCAAGTCTCAGTACAGGAA |
| CUX2 | CGAGACCTCCACACTTCGTG | TGTTTTTCCGCCTCATTTCTCTG |
| AGAP1 | ACACGAGCGAGATTCCTCTG | CACCGTTTCAGGTCGTTGGA |
| NLGN2 | TGGTTCACCGACAACTTGGAG | GCACGTAGAGGTTGAGGTACAG |
| LRP1 | AACGAGCATAACTGCCTGGG | CGTACACTGAGCACTCATCAAA |
| MEGF6 | GTGCTCCTGCCACAACAAC | CAGCCCTCTCCAAATGAACC |
| PDGFRB | AGACACGGGAGAATACTTTTGC | AGTTCCTCGGCATCATTAGGG |

**TABLE S3 Antibodies in this work.**

| Antibody | Source | Identifier |
| --- | --- | --- |
| GAPDH | Proteintech | Cat#10494-1-AP |
| H3K27ac (ChIP) | Cell Signaling Technology | Cat#8173 |
| H3K4me1 (ChIP) | Cell Signaling Technology | Cat#5326 |
| BRD4 (ChIP, WB) | Cell Signaling Technology | Cat#13440 |
| IGF2BP2 (RIP, WB ) | Proteintech | Cat#11601-1-AP |
| IGF2BP3 (RIP, WB) | Proteintech | Cat#14642-1-AP |
| CDK7 (WB) | Proteintech | Cat#27027-1-AP |
| DDX21 (Co-IP, ChIP, WB) | Proteintech | Cat#10528-1-AP |
| Caspase3 (WB) | Proteintech | Cat#19677-1-AP |
| PARP (WB) | Cell Signaling Technology | Cat#9532 |
| ULK1 (WB) | Cell Signaling Technology | Cat#8054 |
| YBX1 (Co-IP, ChIP, WB) | Proteintech | Cat#20339-1-AP |
| Flag | Sigma-Aldrich | Cat#F3165 |
| METTL3 | Abcam | Cat#ab195352 |
| METTL14 | Abcam | Cat#ab309096 |
| FTO | Abcam | Cat#ab124892 |
| ALKBH5 | Sigma-Aldrich | Cat#HPA007196 |

| **TABLE S4 The list of 123 SE-related genes in AML.** | | | | | | |
| --- | --- | --- | --- | --- | --- | --- |
| ADRBK1 | CDKN1B | EFCAB2 | IGF2BP2 | MBP | SELPLG | ZFP36 |
| AFF1 | CDKN2C | ELF1 | IGF2BP3 | MEF2C | SENP6 | ZMIZ1 |
| ANP32A | CELF2 | ERLIN1 | IRX3 | MEF2D | SLC25A13 | ZNF521 |
| ANXA4 | CITED2 | ETV6 | ITGAL | MEIS1 | SLC39A10 | IRF2BP2 |
| ARHGAP4 | CLIC1 | F3 | JARID2 | MIR223 | SNHG3 | CDK6 |
| ARHGEF2 | CNOT6L | FLT3 | JUNB | MIR23A | SNORA14B | EEF1A1 |
| ARID1A | CPEB2 | GFI1 | JUND | MIR650 | SPN | MAP3K1 |
| ARID2 | CRADD | GNA15 | KAZALD1 | MYB | SREBF1 | SATB2 |
| ATP8B4 | CTDSP1 | HIST1H1C | KLF16 | NEAT1 | SRGN | XBP1 |
| AZIN1 | DACH1 | HIST1H2BD | LAIR1 | PCDHGC5 | SSBP4 | C10ORF91 |
| AZU1 | DAD1 | HIST1H4B | LAT2 | PLEKHO1 | TARBP1 | E2F3 |
| BAHCC1 | DDIT4 | HLX | LCP1 | PTMA | TBC1D5 | ID2 |
| BARX1 | DENND2D | HMX2 | LOC100271722 | PTPN7 | TBCC | MALAT1 |
| BCL11A | DKFZP686O1327 | HNRNPF | LOC284648 | RASA3 | TMEM105 | SATB1 |
| BCOR | DLEU2 | HNRNPL | LOC648987 | RCSD1 | TNRC18 | USP4 |
| BLM | DNAJB12 | HNRNPU | LPHN2 | REEP3 | TTYH3 |  |
| BMI1 | DUSP10 | HOMER3 | LY86 | RREB1 | UBASH3B |  |
| BRD2 | DUSP6 | ICOSLG | LYST | RUNX2 | USP31 |  |

| **TABLE S5** The clinical characteristics of AML patients based on DDX21 expression. | | | |
| --- | --- | --- | --- |
| Variables | DDX21 expression | | *P* Value |
|  | Low (N=51) | High (N=98) |  |
| Age, median (IQR), years | 61 (51-68) | 55 (42.75-66) | 0.078 |
| Sex |  |  | 0.210 |
| Male, n (%) | 31 (60.8%) | 49 (50.0%) |  |
| Female, n (%) | 20 (39.2%) | 49 (50.0%) |  |
| FAB classification |  |  | 0.437 |
| M0, n (%) | 6 (11.8%) | 7 (7.1%) |  |
| M1, n (%) | 10 (19.6%) | 23 (23.5%) |  |
| M2, n (%) | 11 (21.6%) | 24 (24.5%) |  |
| M3, n (%) | 8 (15.7%) | 5 (5.1%) |  |
| M4, n (%) | 10 (19.6%) | 23 (23.5%) |  |
| M5, n (%) | 5 (9.8%) | 12 (12.3%) |  |
| M6, n (%) | 0 (0.0%) | 2 (2.0%) |  |
| M7, n (%) | 1 (1.9%) | 2 (2.0%) |  |
| WBC, median (IQR), (×10^9^/L) | 13 (2-50) | 17.50 (5.00-47.25) | 0.996 |
| HB, median (IQR), (g/L) | 100 (90-100) | 90.0 (90.0-102.5) | 0.572 |
| PLT, median (IQR), (×10^9^/L) | 45 (25-90) | 49.00 (29.75-87.25) | 0.944 |
| Cyto_Risk |  |  | 0.974 |
| Low, n (%) | 10 (19.6%) | 19 (19.4%) |  |
| Intermediate-high, n (%) | 41 (80.4%) | 79 (80.6%) |  |

| **TABLE S6** The detailed information of public datasets we utilized in the study. | | | | | | |
| --- | --- | --- | --- | --- | --- | --- |
| **Datasets** | **Source** | **Sample** | **Sample size** | **Experiment type** | **Purpose** | **Citation** |
| GSE65161 | GEO | MOLM14 cell | / | ChIP sequencing | To find super-enhancer-associated genes. | PMID: 26416749 |
| GSE90639 | GEO | HEK293T cell | / | RIP sequencing | To identify the mRNA targets of the insulin-like growth factor-2 (IGF2) mRNA-binding proteins 1, 2, and 3 (IGF2BP1/2/3). | PMID: 29476152 |
| GSE94613 | GEO | MOLM13 cell | / | MeRIP sequencing | To investigate the genes modified by m^6^A modification. | PMID: 33902106 |
| GSE78827 | GEO | MV4-11 cell | / | RNA sequencing | To search the transcripts inhibited by JQ1. | PMID: 27757418 |
|  |  | OCI-AML3 cell | / |  |  |  |
| GSE114868 | GEO | AML patients | 194 | RNA sequencing | To compare the gene expressions between AML patients and the healthy controls. | PMID: 31234830 |
|  |  | healthy donor | 20 |  |  |  |
| GSE37642 | GEO | AML patients | 553 | RNA sequencing | To establish gene expression profiling of AML patients. | PMID: 29242298  PMID: 24923295  PMID: 23382473  PMID: 26674118 |
| GSE175712 | GEO | MOLM13 cell | / | ChIP sequencing | To explore the targets bound by YBX1. | PMID: 34465866 |
| GSE56802 | GEO | HEK293T cell | / | ChIP sequencing | To explore the possible binding sites of DDX21 on ULK1. | PMID: 25470060 |
| GSE1159 | GEO | AML patients | 285 | RNA sequencing | To analyze the gene expression profiling of AML patients and normal controls. | PMID: 17910043  PMID: 15084694 |
|  |  | Normal controls | 8 |  |  |  |
| TCGA-LAML | TCGA | AML patients | 173 | RNA sequencing | To establish gene expression profiling of adult AML patients. | / |
| TARGET-AML | TARGET | AML patients | 142 | RNA sequencing | To establish gene expression profiling of child AML patients. | / |
